# Social influences complement environmental cues to stimulate migrating juvenile salmon

**DOI:** 10.1186/s40462-026-00644-y

**Published:** 2026-04-06

**Authors:** Maria Kuruvilla, Thomas P. Quinn, Joseph H. Anderson, Mark D. Scheuerell, Erika M. Miller, Andrew G. Berger, Connie Okasaki, John R. McMillan, George R. Pess, Peter A. H. Westley, Andrew M. Berdahl

**Affiliations:** 1 Department of Biology, University of Victoria, Victoria, BC V8P 5C2 Canada; 2 Quantitative Ecology and Resource Management, University of Washington, Seattle, WA 98195 USA; 3 School of Aquatic and Fishery Sciences, University of Washington, Seattle, Washington, 98195 USA; 4 Washington Department of Fish and Wildlife, Post Office, Box 43200, Olympia, Washington, 98504-3200 USA; 5 U.S. Geological Survey Washington Cooperative Fish and Wildlife Research Unit, School of Aquatic and Fishery Sciences, University of Washington, Seattle, Washington, 98195 USA; 6 Marine Biology, University of Washington, Seattle, WA 98195 USA; 7 Puyallup Tribe Fisheries Department, 6824 Pioneer Way E., Puyallup, WA 98371 USA; 8 The Conservation Angler, 16430 72nd Ave W., Edmonds, WA 98026 USA; 9 George Pess Consulting, LLC 10333 44th Ave NE, Seattle, WA 98125 USA; 10 Department of Fisheries, College of Fisheries and Ocean Sciences, University of Alaska Fairbanks, Fairbanks, AK USA

## Abstract

**Background:**

The large-scale seasonal migrations undertaken by many species require complex navigational and timing decisions. Animals migrating in groups might benefit from collective decision making, especially if the environment has large local variation rather than smooth gradients in, for example, salinity or temperature, or is unpredictable, or if the migrants cannot rely on individually acquired information. We focus on juvenile salmon whose downstream migration is timed to match suitable conditions for growth and survival at sea. While the environmental and physiological factors that influence the timing of migration have been well studied, the influence of social interactions on migration timing is poorly understood.

**Method:**

We compiled data on two species of juvenile salmon, collected at traps over 19 years, during their downstream seaward migration in three rivers in Washington state along with relevant environmental data. We developed state space statistical models to estimate the influence of hatchery-produced salmon in stimulating the downstream migration of wild salmon, while also incorporating potential environmental stimuli.

**Results:**

Our results are consistent with the “pied-piper” hypothesis that large numbers of migrating hatchery-origin salmon provide a social cue stimulating migration of co-occurring wild salmon. The increase in the number of hatchery salmon counted at the trap was a strong predictor of the increase in wild sub-yearling Chinook salmon in the Dungeness, Puyallup, and Skagit rivers and yearling coho salmon in the Puyallup and Skagit rivers. Migration timing was also associated with abiotic factors related to temperature, river flow, and time of year.

**Conclusions:**

Our findings highlight the potential for social cues to affect migration timing of downstream migrating salmon, in concert with environmental factors. Incorporating social information into timing decisions may allow animals to benefit from collective decision-making strategies and better time their migrations. Moreover, understanding the effects of large-scale hatchery releases on wild salmon migration may provide valuable insights for planning the timing and duration of hatchery releases.

**Supplementary Information:**

The online version contains supplementary material available at 10.1186/s40462-026-00644-y.

## Introduction

From monarch butterflies (*Danaus plexippus*) to blue whales (*Balaenoptera musculus*), many species undertake large-scale migrations to take advantage of seasonal changes in abiotic and biotic conditions [1]. These migrations are often challenging in terms of energetics, orientation and navigation, and timing. Migrations are often group activities, and collective decision-making – where individuals adjust their behavior based on the collective behaviors of the larger group – may improve the accuracy of orientation and homing [2, 3]. For example, the homing efficiency of homing pigeons (*Columba livia*) improved when flying in flocks [4, 5]. Even migrations that appear to be asocial – such as the highly dispersed migrations [6] of monarch butterflies [7] or blue whales [8] – may be influenced by social information, through visual or olfactory cues or long-range communication [9, 10]. While most of the research on collective decision making during migration has been focused on deciding where to go (i.e., collective navigation), interest is expanding to include another critical question migrants face: when to go [11, 12].

Migration timing decisions must often be made based on locally variable information only weakly correlated with conditions in the animals’ destinations [13, 14]. For example, migrating East Atlantic light-bellied brent goose (*Branta bernicla hrota*) failed to match the shifting timing of spring conditions in the Arctic because conditions experienced in the distant winter staging area did not shift [15]. Some animals can learn from previous experience [16] but first-time migrants may not be able to determine conditions in the destination until they arrive, and in some species they cannot follow their parents or other experienced migrants. Anadromous Pacific salmon (*Oncorhynchus* spp.) migrate as juveniles from freshwater habitats to the ocean for feeding, and return as adults to their natal site to spawn [17]. The juvenile salmon have not been to sea before, their parents died after spawning months before, and there is no way to obtain information about conditions at sea prior to departing. Most species spend months or years feeding in freshwater habitats before migrating, though some migrate immediately or almost immediately to sea after emerging from gravel nests [17]. Chinook salmon (*O. tshawytscha*) migrate to sea in their first year of life, shortly after they emerge from the gravel or after a few months of feeding in streams, or the following spring after feeding for a full year in the stream [17]. Coho salmon (*O. kisutch*) typically migrate to sea after one or two years in streams, though fall downstream movement within rivers is common and some enter marine waters then. As part of the transformation from stream-resident parr to seaward migrating, saltwater-tolerant smolts, there is a shift from territorial behavior and upstream orientation to more aggregative behavior and downstream swimming in Pacific salmon [18] and the related Atlantic salmon, *Salmo salar*, and brown trout, *S. trutta* [19].

The smolt migration to sea is timed, on a broad level, to match the optimal conditions for growth and survival in estuaries and at sea [14, 20, 21]. These conditions, including sea surface temperature, spring plankton bloom and predator distributions, vary from year to year [22, 23] so the optimal dates for marine entry vary among sites and years. Additionally, there are species level differences in optimal dates for migration. For example, higher survival was associated with earlier migration in Chinook salmon but later migration in hatchery coho salmon [24, 25]. Photoperiod, combined with internal circannual rhythms, provides the primary cue for the complex changes in physiology and endocrine systems needed to prepare the fish for the radical change in osmotic environments [26, 27]. The behavioral changes from upstream-oriented territorial parr to downstream-migrating, more aggregative or schooling smolts [28] are less well known than the physiological processes [18]. Recent direct observations, however, revealed active schooling by smolts in a river [19], thus schooling is an important component of migratory behavior. In this case there were inter-specific differences in the proportion of fish schooling - higher in Atlantic salmon than brown trout, indicating the importance of comparative studies.

The importance of salmonids in commerce, culture, and ecosystems in the northern Pacific Ocean and Atlantic Ocean [29] has led to many long-term monitoring programs to assess the abundance of seaward migrating smolts and returning adults [30], and efforts to determine the environmental stimuli that trigger their movement [31–33]. Daily counts of migrating juvenile salmonids from traps typically show a series of peaks and troughs rather than the smooth, more or less bell-shaped curve seen when data from many years are pooled. Efforts to model these day-to-day patterns have tended to focus on abiotic features of the rivers and especially aspects of water temperature, river flow, and lunar phase, typically with only modest success [34]. Considerable variation is left unexplained after various combinations of abiotic data are included in models. However, this fine-scale variability in count data from the spawning migrations of adult salmonids have been replicated by models that include social behavior [35]. Therefore, we predict social interactions may also be important in the timing of migrations of salmonid smolts. Specifically, downstream migration by conspecifics may motivate movement by other individuals in the stream who are physiologically ready to migrate but without such stimulation by migrants might not migrate until later that season.

Hatcheries, used for many decades with the intention to bolster salmon populations, may incidentally provide a way to test for the use of social information in the migration timing decisions of juvenile salmon. Hatcheries rear young salmon in mortality-reduced conditions, before releasing them into rivers where they migrate to the ocean. Hatcheries often release, all at once or over a narrow period, many thousands of juveniles with a more uniform size range and physiological readiness to migrate compared to fish rearing naturally. These releases can greatly increase the number of fish in the rivers over brief periods, relative to the stream’s natural production. Data from traps reveal that hatchery-produced salmon often migrate rapidly, and *en masse*, to the ocean (Figs. 2 and 3). The hatchery salmon cannot migrate before they are released and are usually very numerous, creating an asymmetry between natural-origin and hatchery-origin salmon in terms of opportunity to migrate on a given date, and abundance. Trap operators and biologists have hypothesized that these large numbers of hatchery-origin fish may provide a social cue to migrate for the wild conspecifics (personal communication with Peter Topping, Washington Department of Fish and Wildlife), which typically have a much more protracted migratory window [36–38]. Efforts to test this so-called “pied-piper” hypothesis (referring to the folktale of the pied piper of Hamlin) have yielded supportive [34] or equivocal results [39].

With respect to migrating juvenile salmon, the pied piper hypothesis proposes that fish that are approximately physiologically ready, but have not yet begun to migrate, may detect conspecifics migrating downstream past them through visual, odor or lateral line cues. Due to social attraction, the former may be influenced to join the latter, increasing the social migration stimulus for fish further downstream. Each wave of emigrating smolts tends to take with it those that are most physiologically ready to migrate, leaving those that are less prepared to migrate. The system may then exhibit a refractory period, with few fish leaving, even with ideal abiotic conditions, until the remaining fish increase in readiness and the process repeats itself until the population has all migrated. This conceptual model of migration in wild populations thus incorporates social with abiotic factors triggering migration, the result being a migration characterized by pulses of migrants even in the presence of smoothly varying potential environmental triggers for migration. In the presence of stimulatory physical conditions, the social interactions are predicted to further elevate the number of migrants. The null hypothesis is that wild salmon only respond to environmental factors and do so independently of conspecifics.

In this paper, we leverage a multidecadal dataset to test for the use of social information in the timing decisions of migrating salmon smolts of two different species. Specifically, we use a state space statistical model to estimate the influence of hatchery-produced coho and Chinook salmon smolts on the movement decisions of wild-origin smolts, relative to established abiotic factors affecting movement (Table 1). A state space model allows us to estimate the effect of covariates (both environmental and social) on the number of wild salmon migrating after accounting for process errors, observation errors, and autocorrelation. Chinook and coho salmon are widely propagated in the region’s hatcheries [40], and transition from stream-resident to migratory behavior after several months to a year [17], and thus are suitable for testing our hypothesis. While augmenting our view of social decision making to include temporal aspects of migration, this work also contributes to our knowledge of salmon life history and our understanding of the unintended ecological impacts of hatcheries on wild salmon populations [41, 42].

**Table 1:** List of environmental covariates, source of data, description, data transformation, predicted effect on number of wild salmon migrating (positive or negative) of the covariates used in MARSS analysis for each river. The reference column provides an example of a paper that has used the given covariate in a model of juvenile migration timing of Chinook, and coho salmon in various rivers. (–) indicates no reference but we hypothesize that the covariate would have an effect on migration timing of juvenile salmon

Covariate	Data Source	Description	Transform-ation	Predicted effect (+/-)	Reference
Temperature anomaly	WA Department of Ecology, WDFW, USGS, USDA Natural Resources Conservation Service	Daily mean of water temperature (Skagit River) or water temperature measured at a time point closest to the midpoint of the daily trapping duration (Dungeness River) or daily mean of air temperature (Puyallup River) minus the moving average of temperature calculated for 32 days prior.	Scaled and centered	+	[25, 31, 48]
Season	Calculated	Difference between the day of year and the day of year that 50% of the salmon migrated that year.	Scaled	+	–
Temperature difference	Calculated from WA Department of Ecology, WDFW, USGS, USDA Natural Resources Conservation Service data	Change in water/air temperature between that day and previous day $\:{x}_{t}-{x}_{t-1}$. Daily mean of water temperature (Skagit River) or water temperature measured at a time point closest to the midpoint of the daily trapping duration (Dungeness River) or daily mean of air temperature (Puyallup River)	Scaled	+	[48]
Flow anomaly	WA department of Ecology, USGS	Daily mean of water flow (Skagit River), or daily flow measured at a time point closest to the midpoint of the trapping duration (Dungeness River), or daily flow measured when the trapping started (Puyallup River) minus the moving average of flow calculated for 32 days prior.	Scaled and centered	-	[47]
Flow difference	Calculated	Change in water flow between that day and previous day $\:{x}_{t}-{x}_{t-1}$	Scaled	+	[48]

**Fig. 1:**
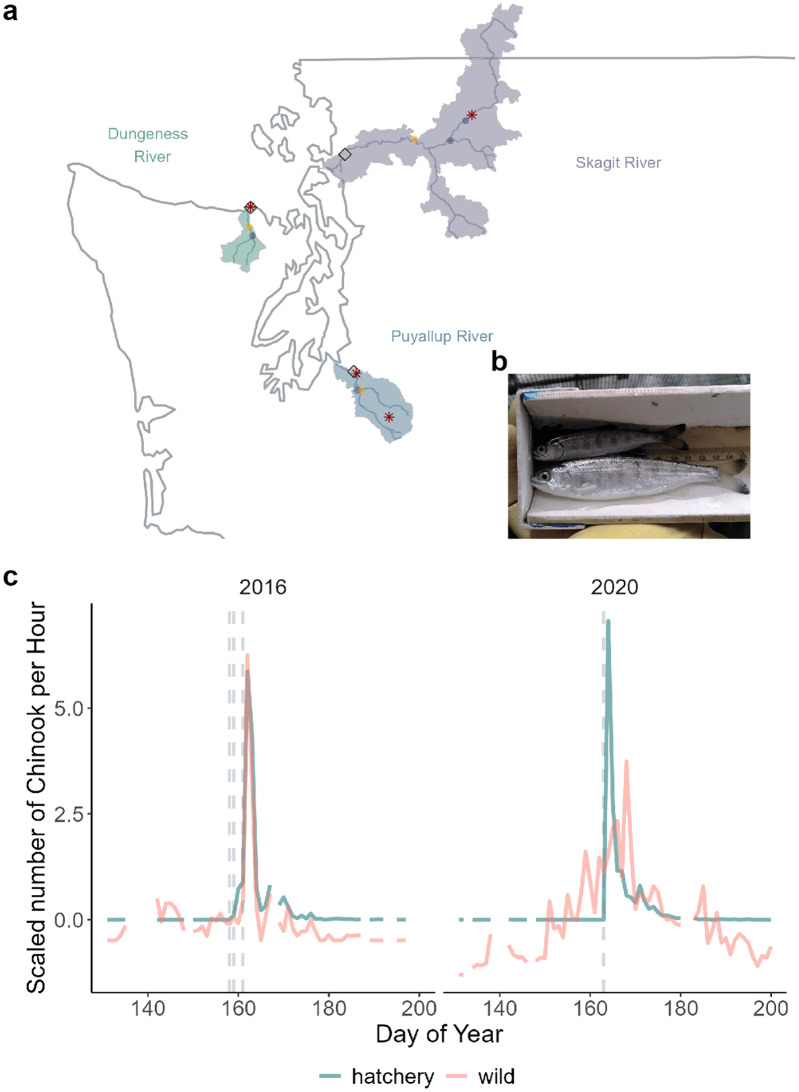
**a**) Map of the rivers (lines) included in this study, along with their watersheds (shaded regions) in Washington State, USA. Black diamonds indicate the location of the smolt traps. Purple and orange circles indicate common Chinook and coho salmon release sites respectively. Red stars indicate environmental data sites. **b**) Image of a wild (top), and a hatchery (bottom) yearling coho salmon caught in the Dungeness River trap. **c**) Scaled values of number of Chinook salmon per hour caught in the trap in the Dungeness River from 2016 and 2020. Gray vertical dashed lines indicate the date of hatchery release(s). In some years (e.g., 2016), many wild salmon migrate past the trap concurrent with the pulse of hatchery fish. In other years (e.g., 2020), this correlation is not clear

## Methods

### Trap data

To estimate the influence of hatchery salmon on the migration of wild salmon, we focused on three rivers in Washington state with hatchery programs propagating sub-yearling Chinook salmon (hereafter Chinook salmon) and yearling coho salmon (hereafter coho salmon) (Table S1). We refer to hatchery-origin salmon as hatchery salmon and natural-origin salmon as wild salmon, acknowledging that fish termed “wild” may have recent hatchery ancestry (i.e., hatchery-origin parents spawning in the river). Other life history variants such as yearling Chinook salmon, sub-yearling coho salmon, and steelhead trout (*O. mykiss*) smolts, are much less numerous and were excluded from our analyses. We used data from salmon traps deployed in the Dungeness (years 2005–2020), Puyallup (years 2004–2021), and Skagit (years 2010–2022) rivers to quantify juvenile salmon populations migrating to sea. These three rivers are representative of those where coho and Chinook salmon naturally occur in the region, with a range of attributes. The Skagit is the largest in terms of watershed area and volume, followed by the Puyallup, and the Dungeness is the smallest (Fig. 1a). The extent of human habitat modification is greatest in the Puyallup River. We chose these rivers because they have long-term records of migrating hatchery and natural origin salmon and because we sought to test the hypothesis in a range of settings. Average number of releases per year and average number of salmon released per year in each river are shown in Table S16.

Migrating smolts were caught in a single floating screw trap (in the Dungeness and Puyallup rivers) and a floating scoop trap and a floating screw trap (in the Skagit River), counted and released [43]. The hatchery origin salmon were identified by trained staff operating the traps by the presence of an internal coded wire tag (detected magnetically), or a clipped adipose fin. While most hatcheries try to mark all the salmon that are released in the rivers, a small percentage as estimated and reported in the Regional Mark Information System (RMIS) database by the hatchery (< 5% of hatchery coho salmon in the Dungeness and Skagit rivers; <5% of hatchery Chinook salmon in the Skagit and Puyallup rivers; <12% of hatchery Chinook salmon in the Dungeness River (24 out of the 27 releases had < 5%); <3% of hatchery coho salmon in the Puyallup River except for one instance (2021) when 63%) were unmarked (unclipped and untagged). We assumed that the percentage of unmarked hatchery salmon released from the hatchery is the same as the percentage of unmarked hatchery salmon in our much smaller recapture sample. The unmarked hatchery salmon are comparable in abundance to the wild salmon present in the Dungeness and Puyallup rivers. Because hatchery salmon are often bigger than wild salmon and have a different appearance (Fig. 1b), the trap operators sometimes identify ‘unmarked hatchery’ fish based on body size, particularly following recent hatchery releases; however, this categorization may not always be accurate. In the Puyallup River, where Chinook salmon were the primary driver of the monitoring project, the unmarked hatchery Chinook salmon were accounted for in the years 2016–2021 prior to data sharing by subtracting their number, estimated by the RMIS database, from the number of wild salmon caught. We used the untagged hatchery release numbers from the RMIS database to investigate the issue of misidentification of unmarked hatchery salmon as wild salmon at the traps in all the rivers by using data from RMIS. We used the proportion of unmarked hatchery salmon released by the hatcheries to look at the correlation between the estimated number of unmarked hatchery salmon released and the number of wild salmon caught in the traps on the day with the peak number of hatchery salmon within 10 days of hatchery release (Figure S2). A strong relationship would suggest wild catch totals were mostly driven by unmarked hatchery fish, potentially confounding our counts of wild salmon. For Chinook salmon in the Puyallup River, we found that correlation between the number of unmarked hatchery salmon released and the number of wild salmon subsequently caught in the trap (0.35) was dominated by the release in 2019. Our estimates of the covariates in the best model did not change when we excluded 2019 from our analysis (Figure S21), and we note that the data were already corrected for unmarked hatchery fish. For coho salmon in the Puyallup River, since a large number and a high proportion of unmarked hatchery coho salmon were released in 2021, we ran our analysis without the data from 2021 and found that our results remained unchanged (Figure S22). For coho salmon in the Skagit River, we found a moderate correlation (0.36) in the number of unmarked hatchery salmon released and the number of wild salmon subsequently caught in the trap. However, since the number of wild coho salmon caught in the trap is comparable to the total number of hatchery salmon caught in the trap in the Skagit River, and only a very small (always < 5% in our dataset) percentage of the hatchery salmon are unmarked, it is unlikely that the correlation between unmarked hatchery salmon and wild salmon is driven by misidentification. We confirmed that unmarked hatchery coho salmon in the Skagit River did not influence our result by performing a sensitivity analysis, in which we relabeled known hatchery fish as wild and reran our statistical analysis on those synthetic data. We used the rate of increase in apparent pied piper effect with mislabeled data to project backward and estimate the measured effect of hatchery difference if we were able to correct currently mislabelled fish (Figure S20).

In all three rivers, some Chinook salmon migrate shortly after emergence in January to March (fry migration) and others migrate later, from May to July, after feeding in the river for several months (parr migration), as is typical in the region [44, 45]. This results in a strongly bimodal distribution of migration times. Since the hatchery smolt releases coincided with the later parr migration, we restricted our analysis to days of the year 130–200 in the Dungeness River and days of the year 130–218 in the Puyallup River (Figure S1). In the Skagit River, the proportions of fry migrants and parr migrants vary from year to year [46]. Most of the sub-yearlings migrated as fry in the years of data we had, leaving only a small proportion of the migrants remaining during hatchery releases. The Skagit River trap was only operated up to day 189 in some years. Therefore, we used only data from days 150–189 for Chinook salmon in the Skagit River. For coho salmon, we used data between days 120 and 160 in the Dungeness River, between days 90 and 160 in the Puyallup River, and between days 100 and 150 in the Skagit River.

In the Dungeness and Puyallup Rivers, salmon caught in the trap were counted roughly twice a day – at around 06:00 and 18:00. The counts made at around 06:00, with the trap operating between 18:00 and 06:00, are hereby called ‘night’ counts even though we acknowledge that the trap was operating during some daylight hours. Similarly, the counts made at 18:00 are hereby called ‘day’ counts. ‘Night’ counts were typically much higher than the ‘day’ counts (Figure S3) because the juvenile salmon tend to migrate at night. Since very few coho salmon, and a very small proportion, migrated during the day in the Dungeness River (proportions shown in Figure S3), we only analyzed the ‘night’ counts for this population. In the Skagit River, the trap operated only during the nights and counted at around 06:00, except for every third day, when the trap operated all day, but was checked twice at around 06:00 and 20:00. Because two thirds of the ‘day’ counts were not enumerated, we only analyzed the ‘night’ counts in the Skagit River. The counts were separated by scoop trap counts and screw trap counts in the Skagit River. The scoop trap counts and the screw trap counts were analyzed separately. In all rivers, because the time at which the trap started and stopped operating varied slightly each deployment, we used the time deployed to calculate the catch per unit effort or catch rate for each day or night by dividing by the number of hours deployed.

The number of wild smolts caught in the trap each day was our response variable. We log-transformed this number (after adding 1 to each data point to avoid infinite values) because the data were log normally distributed, as determined visually. We scaled (divided by the standard deviation) and centered (subtracted the mean) the data for each year to account for large year-to-year variation in catches so that we could compare among the years.

The difference between the number of hatchery smolts caught in the trap on successive days was our social covariate. We used the difference, rather than the absolute count, of hatchery fish to match our process model (Eq. 2). Because the state process models day-to-day changes in wild smolt abundance, we used day-to-day changes in hatchery smolt catches as the covariate, allowing transient hatchery releases to affect short-term wild smolt dynamics without implying cumulative effects after releases end. We scaled, but did not center, the difference in the catches of hatchery salmon counts caught on consecutive days so that the difference in counts remained 0 when there was no change in the number of hatchery fish. Within 10 days after a hatchery release, the traps sometimes got inundated with hatchery salmon. To avoid this, trap operators in all three rivers sometimes suspended operations for a few days, resulting in missing data. We linearly interpolated the data on hatchery fish for days when the trap was out since the analysis that we used does not allow missing values in the covariates. We interpolated around 16% (526 values) of the data from the Dungeness River, 37% (1148 values) of the data from the Puyallup River and 13% (267 values) of the data from the Skagit River. Most of the missing values in the Puyallup and Skagit rivers were towards the end of the season and during the Covid-19 lockdown in 2020. Because missing values are allowed in the response variable of our model, we did not interpolate missing values of the wild salmon counts.

**Fig. 2:**
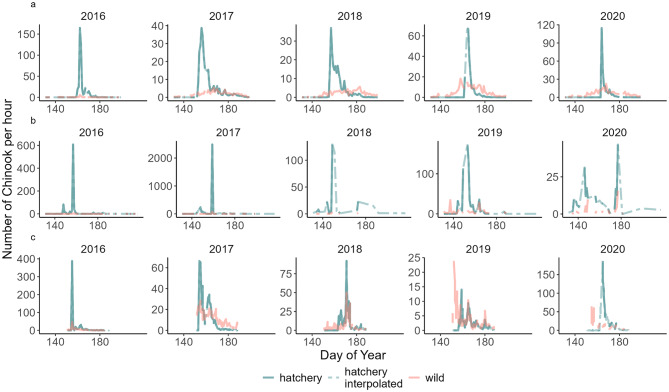
Daily number of Chinook salmon caught in the trap at night for 2016–2020 in **a**) Dungeness, **b**) Puyallup, and **c**) Skagit rivers

**Fig. 3:**
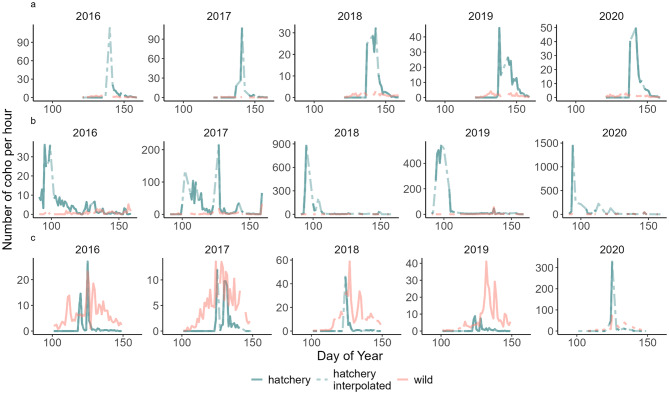
Daily number of coho salmon caught in the trap at night in 2016–2020 in the **a**) Dungeness, **b**) Puyallup, and **c**) Skagit rivers

### Environmental data

We aimed to estimate the social effects on the migration of salmon smolts, while controlling for environmental effects, which may cause many wild and hatchery fish to independently leave the same day. Based on previous studies [31, 47–49], we selected environmental covariates: which have been reported to influence daily Chinook [31, 47, 49] and coho salmon smolt migration [48, 49] in British Columbia [47, 49] and Oregon [31, 48]. Specifically, for our analysis, we selected flow anomaly, flow difference, temperature anomaly, temperature difference, and season (Table 1). Although this is not a comprehensive review of the literature on environmental effects on salmon smolt migration, and the effects of environmental cues on migration timing might differ due to geographic variations [48], we predicted that these covariates would affect the daily downstream migration of Chinook and coho salmon smolts. All the environmental data that we used were collected in the same river basin as the migrating salmon. While these data do not capture the full range of environmental conditions that the fish experience throughout the spatial extent of juvenile rearing habitats, we assumed that measured water/air temperature and discharge downstream were correlated to the water temperature and discharge experienced by the fish.

We used flow data from the Washington Department of Ecology (Dungeness River mouth, Station ID 18A050) for the Dungeness River, from the USGS (Newhalem, Site no. 1217800) for the Skagit River, and the data from USGS (Alderton site, Site no. 12096500) for the Puyallup River. For the Skagit River, we obtained water temperature data from USGS (Newhalem, Site no. − 12178000). Water temperature data was not available for the Puyallup River so we substituted this with air temperature data from USDA Natural Resources Conservation Service (Mowich, 941), a site within the Puyallup drainage. For the Dungeness River, we obtained water temperature data from the Washington Department of Ecology (2004–2014, Dungeness River Mouth, Station ID 18A050) and from the Washington Department of Fish and Wildlife (2016–2020). Figure 1a shows the locations of all the sites at which environmental data was collected.

For flow and temperature variables, we used both the change in the value (referred to as ‘difference’) and the departure of the value from a running mean (referred to as ‘anomaly’). To calculate the difference, we found the discrete daily difference (the value on day *i* minus the value of day *i-1*). To calculate the anomaly, we first found the running mean, ${v}^{-}$, by averaging the 32 days leading up to the current day, $\:{{\bar{v}}_{l}}={\sum\:}_{j=i-32}^{i}{v}_{i}$ and then subtracted that value from the value at the current day $\:{{v}_{i}}^{\left(anomaly\right)}={v}_{i}\:-\bar{v\:}$. The anomaly essentially detrends the flow and temperature time series (Figure S4).

We scaled, and for some variables centered, the environmental data for each year to compare the effects of all the covariates (Table 1). Since our analysis did not allow missing variables in the covariates, we linearly interpolated the data for the days in which the data were missing. Before using the environmental data as covariates in the model, we calculated the correlation between every pair of covariates (Figure S5, Figure S10, and Figure S15). If the correlation (rounded to one decimal place) of any pair was > = 0.5, we did not use those covariates in the model at the same time. Instead, we determined the covariate in each pair that had more support from the data (with lower AICc value) and then used those covariates for subsequent analysis.

The migration patterns of salmon roughly follow a hump-shaped curve, with fewer salmon at the beginning, many in the middle, and few at the end of the migration window. Our objective was to study the cues that trigger daily downstream movement of juvenile salmon but we had to account for this non-linear pattern by including the seasonal variable. The seasonal covariate was the day of year subtracted from the day on which 50% of the fish from that population had been caught in the trap or assumed to have migrated (i.e., the median day of migration). The seasonal variable varied linearly with day of year (positive before the median migration day, zero on the median migration day, and negative after the median migration day). Given our modelling framework (logarithm of the difference in smolt counts – described in detail below), a positive association with only this season variable would produce a bell-shaped time series for smolt counts.

### Analysis

We used multivariate autoregressive state-space (MARSS) models to estimate the influence of the number of juvenile hatchery salmon migrating downstream as determined by the difference in the number of juvenile hatchery salmon caught in the trap, and of the different environmental variables, on the number of wild juvenile salmon migrating downstream as determined by the number of juvenile wild salmon caught in the trap [50, 51]. MARSS models allowed us to separate the underlying process from the observed data. In the MARSS framework, $\:{y}_{t}$ is the $\:n\:\times\:1\:$vector of the observations of wild salmon per hour on the $\:t$th day of the year for *n* years and $\:{x}_{t}$ represents the $\:n\:\times\:1$ vector of the true, but unknown number of wild salmon per hour on the $\:t$th day of the year.

They are related by the following equations:

Observation model -

1 $$\:\:{log(y}_{t})=\:log({x}_{t})+{v}_{t},\:{v}_{t\:}\sim\:MVN(R)$$

Process model -

2 $$\:\:{log(x}_{t})=\:log({x}_{t-1})+{Bc}_{t\:}+{w}_{t\:},\:{w}_{t}\sim\:MVN(Q)$$

where $\:{c}_{t\:}$ are the $\:p\:\times\:1$ vector of covariates affecting the states or the average number of salmon per hour migrating, $\:p$ is the number of covariates multiplied by the number of years, $\:B$ is the $\:n\:\times\:p\:$matrix of coefficients relating the effects of the covariates$\:{c}_{t\:}$ to the states $\:{x}_{t}$. $\:{w}_{t}$ and $\:{v}_{t}$ are the $\:n\:\times\:1$vectors of normally distributed errors. $\:R$ is the $\:n\:\times\:n$ variance-covariance matrix of the observation errors and $\:Q$ is the $\:n\:\times\:n$ variance-covariance matrix of the process errors. We assumed that the observations and states were independent between years and that the observation and process variances were the same for all years. Therefore, $\:R$ and $\:Q$ are diagonal matrices.

Since our data for three populations (Dungeness Chinook, Puyallup Chinook, and Puyallup coho) was separated into day and night counts, we fit two different models to the data, with and without equal process variances for day counts and night counts. We hypothesized that the effect of hatchery salmon would be greater during the day because the wild salmon might have stronger visual cues then from the presence of hatchery salmon [33]. Therefore, we allowed the coefficient of the hatchery covariate to be different for ‘day’ and ‘night’ and we predicted that the coefficient would be greater for ‘day’ than for ‘night’. On the other hand, the effect of the environmental covariates was assumed to be equal for ‘day’ and for ‘night’.

To ensure that the errors met the model assumptions, we plotted the residuals of the predicted values against the fitted values (Figures S7, S9, S12, S14, S18, S20), the autocorrelation function of the residuals, as well as quantile-quantile plots. We assumed that both the process errors and observation errors are identical, independent, and normally distributed within years.

We expected the coefficient of the seasonal covariate to be positive for all populations. According to Eq. 2, a positive coefficient for season implies that the number of wild salmon migrating increases before the median day of migration, does not change on the median day of migration, and decreases towards the end of the migration. More generally, a positive estimate of the coefficient of a covariate implies an estimated increase in the difference in the number of wild salmon migrating in the logarithmic scale.

### Model selection and relative importance of variables

Akaike Information Criterion (AIC) is often used to select the best model among models with different combinations of covariates. However, with autocorrelated data, the sample size is much lower than the number of data points we have. Thus, we used AICc, which is AIC with a correction factor for small sample size, to compare models.

We first compared the model with equal process variance for day and night with the model with unequal process variance for day and night and used AICc to select the most appropriate variance structure in all six populations (Tables S2 and S7).

For Chinook salmon in the Dungeness River, two pairs of covariates were positively correlated ( > = 0.5, rounded to one decimal place) with each other: (a) temperature difference and temperature anomaly, and (b) season and flow anomaly (Figure S5). The model with temperature difference was more supported by the data than the model with temperature anomaly and the model with season and the model with flow anomaly has equal support (Table S3). We chose the temperature difference and season covariates for further analysis. For all other populations, none of the covariates were correlated with each other. We then fit each model for each river-species combination with every combination of uncorrelated covariates (including the hatchery covariate) and chose the model with the lowest AICc to get the estimates of the effects ($\:B$) and model diagnostics.

In addition to estimating the best model from model selection, we calculated the relative variable importance as the probability that the given variable is in the best model. We estimated the probability by calculating the Akaike weight of each model and then summing the weights of all the models for which that variable was present. We considered a variable ‘important’ if it has at least 90% probability of being in the best model. While this method does not describe the significance of the variable or the size of the effect that the variable has on the response variable, it describes the relative importance of the covariate in the set of candidate models.

## Results

### Social effects

To test the pied piper hypothesis, we looked at the following three criteria. First, was the hatchery difference covariate in the top model(s) (i.e., did the model with the lowest AICc contain the hatchery covariate)? If so, that would indicate that considering the difference in the number of migrating hatchery fish improved the prediction of wild smolt migration over predictions considering only environmental variables (discussed below). Second, what was the effect size and significance of the hatchery covariate? A positive effect meant that an increase in the number of wild fish was positively correlated with an increase in the number of hatchery fish, even accounting for environmental cues that could cause them both to migrate independently. The significance meant that the estimated range of effect sizes did not include zero. Third, what was the relative importance of the hatchery covariate? The importance measures the role of a variable across all potential model fits and we used a threshold of ≥ 0.9 (has at least 90% probability of being in the best model) to indicate high relative variable importance.

### Chinook salmon

**Fig. 4:**
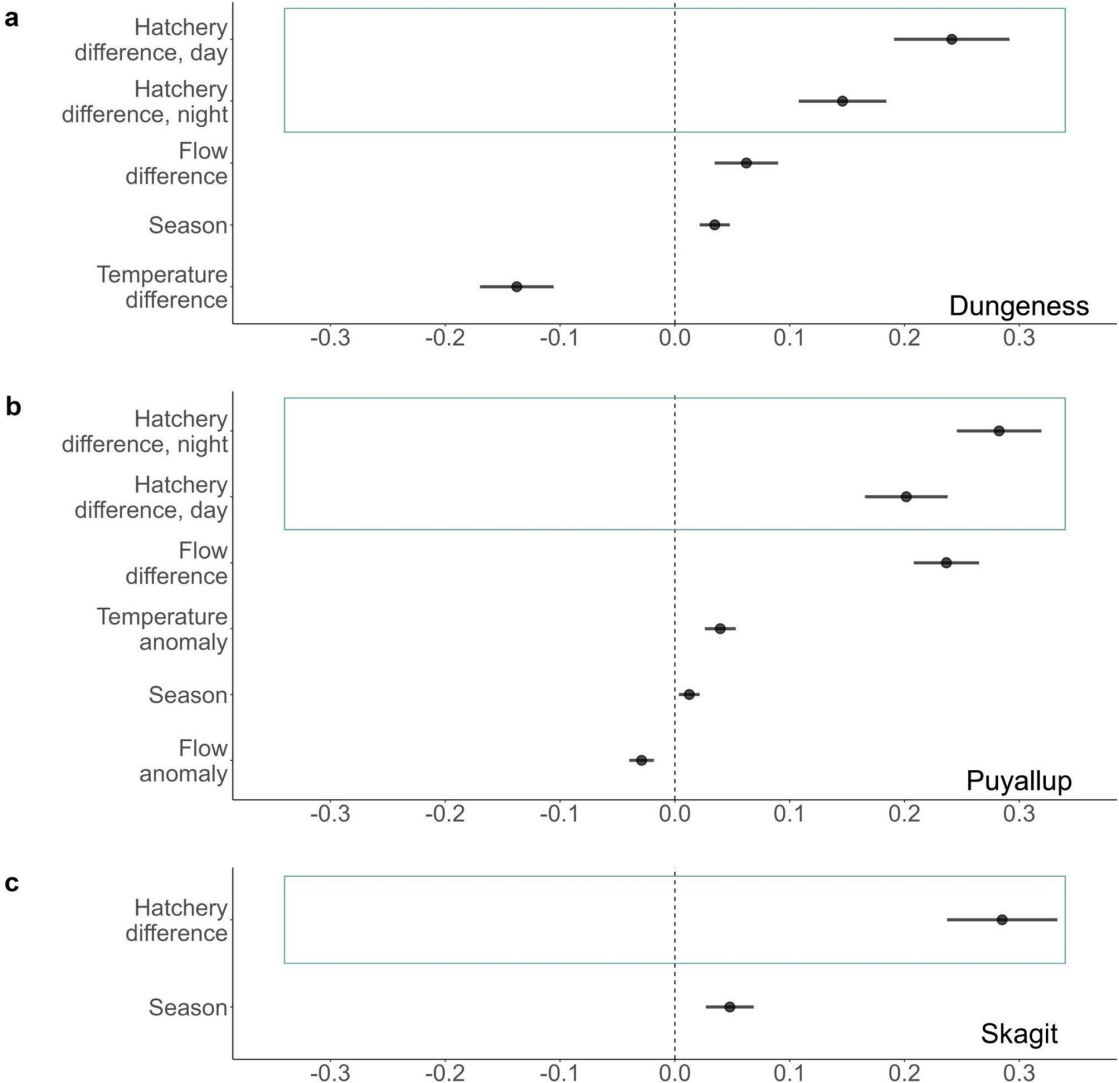
Estimates of the effect of different covariates in the best MARSS model or MARSS model with equal support for the number of wild Chinook salmon in the **a**) Dungeness, **b**) Puyallup, and **c**) Skagit rivers. Blue boxes indicate social variables associated with the pied piper hypothesis

We found support for the pied piper hypothesis for Chinook salmon in the data from all three rivers. The hatchery covariate was included in the top AICc scoring model and the effect of this covariate was positive in all rivers (Fig. 4). In the Dungeness River, the estimated effect of the difference in the number of hatchery fish during the day was higher than the estimated effect at night but in the Puyallup River, the estimated effect at night was higher than the estimated effect during the day. The hatchery covariate was an ‘important’ variable in all three rivers compared to all other variables we tested in model selection (Table S5, S11, S13), as it was in all the top models.

### Coho salmon

**Fig. 5:**
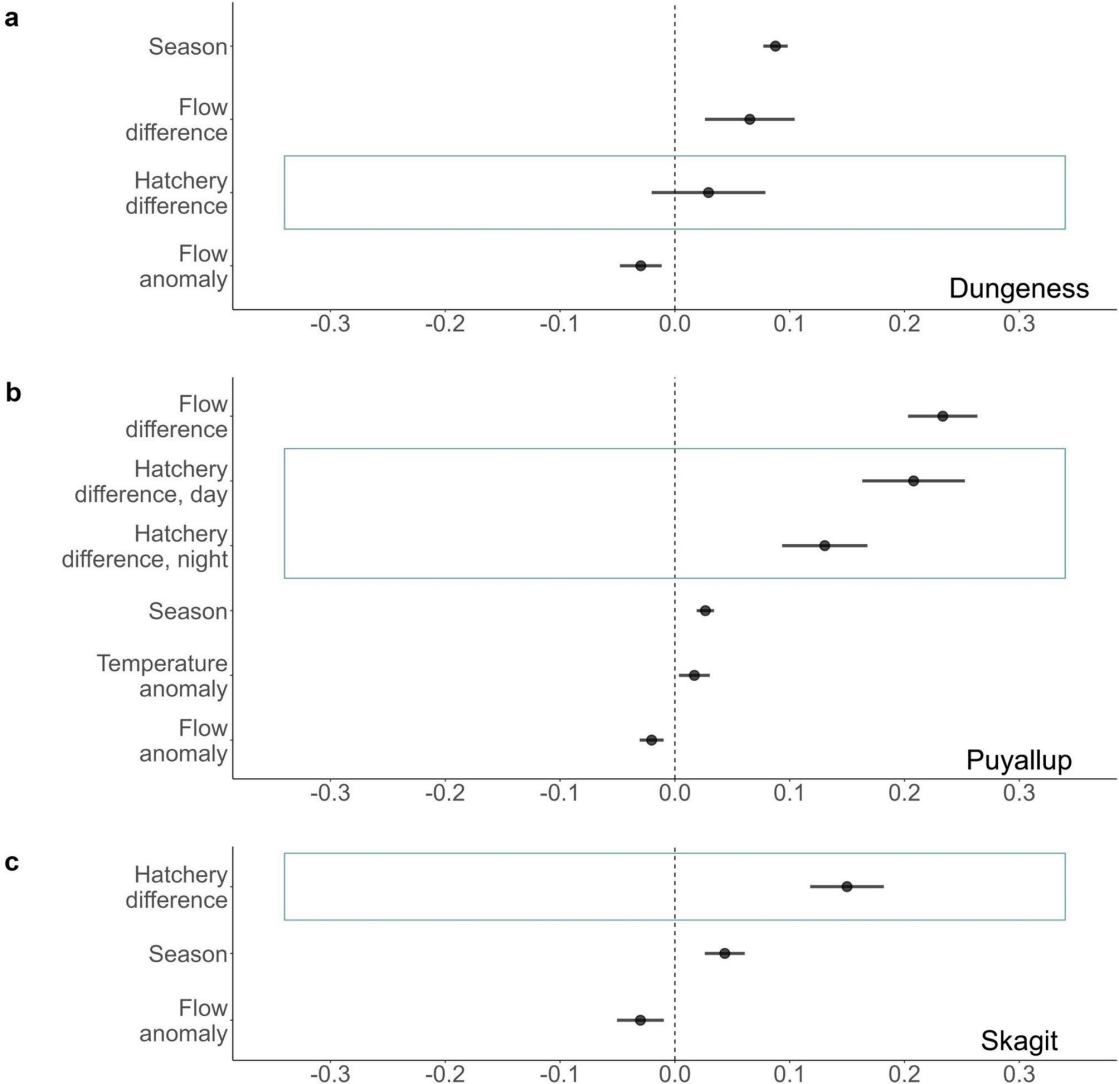
Estimates of the effect of different covariates in the best MARSS model or MARSS model with equal support for the number of wild coho salmon in the **a**) Dungeness, **b**) Puyallup, and **c**) Skagit rivers. Blue boxes indicate social variables associated with the pied piper hypothesis

We found support for the pied piper hypothesis for coho salmon in two of the three rivers. In the Dungeness River, while the hatchery covariate was not in the best model, the model with this covariate had equal support ($\:\varDelta\:AICc<2$, Table S6). The hatchery covariate was not an ‘important’ variable in the Dungeness River compared to all other variables we tested in model selection (Table S5). In both the Puyallup and Skagit rivers the hatchery covariate was included in the top model (Fig. 5b, c). In both rivers, we found a strong positive effect of the hatchery covariate on the number of wild salmon migrating (Fig. 5b, c). In the Puyallup River, the estimated effect of the difference in the number of hatchery fish during the day was higher than the estimated effect of the difference in the number of hatchery fish at night. Further, the hatchery covariate was an ‘important’ variable in both rivers compared to all other variables we tested in model selection (Table S11, Table S13).

### Social summary

Increases in the number of wild salmon migrating coincided with increases in the number of hatchery salmon migrating in five out of the six populations. This social effect was at least as great as the environmental effects explored in the following section and reported in other studies to influence smolt migration. In summary, although our results were correlative, the positive effect of hatchery smolts on the migration of wild smolts was consistent with the results expected under the pied piper hypothesis – wild smolts were stimulated to migrate when they detected many hatchery smolts migrating. In two (Dungeness Chinook and Puyallup coho) out of the three populations with separated ‘day’ and ‘night’ counts, our results were consistent with our prediction that the effect of the hatchery covariate during the ‘day’ was higher than the effect of the hatchery covariate at ‘night’. However, we observed the opposite pattern in Puyallup Chinook.

### Environmental effects

The aim of this manuscript was to test the pied piper hypothesis, therefore we focused on the social variable. However, a shared response to the same environmental variable can be mistaken for social influence, so we also included environmental variables in our models to account for potential common cues shared by hatchery and wild salmon smolts. We focused on environmental cues that may trigger day-to-day fluctuations in the number of migrants rather than continuously varying cues that may regulate development. However, we included a seasonal variable to account for the broad scale migration window. As expected, the seasonal variable was important for all the study populations. Below we report the environmental variables found in the models for our social analysis.

### Chinook salmon

Flow difference – the change in the river’s flow from one day to the next – had a positive effect on the number of Chinook salmon migrating in the Dungeness and Puyallup rivers (Fig. 4a, b) and was classified as an ‘important’ variable in these rivers. That is, increases in flow were associated with increases in salmon migration. However, flow anomaly had a small negative effect in the Puyallup River (Fig. 4b) and was an ‘important’ variable for Chinook salmon in that river. Flow anomaly and difference were not covariates in the top models, nor were they ‘important’ variables for Chinook salmon, in the Skagit River.

Temperature difference – the change in water temperature from the previous day – had a negative effect on the number of Chinook salmon migrating in the Dungeness River (Fig. 4a). This suggests that a drop in water temperature was correlated with more Chinook salmon migrating. Air temperature anomaly had a positive effect on Chinook salmon in the Puyallup river (Fig. 4b), suggesting that more Chinook salmon migrated when the air was warmer.

### Coho salmon

Flow difference had a positive effect on, and was an ‘important’ variable for, the number of wild coho salmon migrating in the Dungeness and Puyallup rivers (Fig. 5a, b). In contrast, flow anomaly had a negative effect on the number of wild coho salmon caught and was an ‘important’ variable for coho salmon in all three rivers (Fig. 5a-c). Air temperature anomaly had a positive effect on, and was an ‘important’ variable for, the number of wild coho salmon caught in the Puyallup River (Fig. 5b).

## Discussion

Our main objective was to determine if social information influences the timing of seaward migration in wild Chinook and coho salmon smolts, in the context of the many abiotic variables known to affect salmon migration (Table 1). Because social interactions among wild fish is difficult to observe directly at this scale, we used migrating hatchery smolts as a focal and quantifiable source of social cues, hypothesizing that migrating hatchery smolts would stimulate wild smolts to move and thereby increase the number of wild smolts migrating above the level expected from contemporary environmental conditions. In five of our six river-species combinations - Chinook salmon in all rivers and coho salmon in the Puyallup and Skagit rivers - the number of hatchery smolts was an ‘important’ variable with a positive statistical relationship with the number of wild salmon smolts, consistent with the pied piper hypothesis. Our results are supported by the visual observation that following hatchery releases of Chinook salmon in the Wenatchee River, wild Chinook salmon smolts moved downstream along with the hatchery smolts unless they could not see the hatchery smolts [39]. Thus, the weight of the evidence indicated an effect of social interactions with hatchery fish, though its magnitude varied among species and rivers, as did the relative effects of different abiotic factors. The findings are also consistent with recent direct observations of schooling by seaward migrating Atlantic salmon and brown trout smolts, and with the difference in schooling tendency between species [19].

For Chinook salmon in all rivers, and coho salmon in the Puyallup and Skagit rivers, the results were consistent with the pied piper hypothesis that the numerous hatchery salmon smolts migrating downstream stimulated the migration of wild salmon smolts. These social influences, however, are best understood in the context of the river’s environmental conditions (aspects of flow and temperature), and time of year (season). In most cases, the effect of the hypothesized social influence was comparable to that of the environmental factors.

Our modelling framework allowed us to address an alternate hypothesis to the pied piper: that hatchery and wild salmon are *independently* responding to the same measured environmental stimuli. For example, if both hatchery and wild smolts independently responded to the river flow, peaks in their respective migration timing distributions would coincide. However, in this scenario, a high number of wild smolts would be correlated with high flow anomaly before the hatchery smolts were released as well as when there was an increase in the number of hatchery smolts migrating. Therefore, inconsistent with our results, this ‘independent response’ model would estimate the coefficient of flow anomaly covariate to be positive and the coefficient of the hatchery difference (difference in the catch rate of hatchery smolts on consecutive days) covariate to be zero and not falsely support the pied piper hypothesis. However, in analyzing fish movement data in a natural environment, we had no ability to control non-social stimuli, and hence could not ensure sufficient contrast among covariates (e.g., flow increases before vs. after hatchery releases) to separate the pied piper vs. independent response hypotheses.

We did not detect a social influence on the migration of coho salmon in the Dungeness River. Several non-mutually exclusive factors relevant to all three rivers may have eroded our ability to detect social cues. First, wild salmon may have been spatially isolated from the hatchery releases, rearing either in tributaries or upstream of the hatchery release sites, thereby diminishing the potential for social interaction. Coho tend to use upstream habitat and the Dungeness River had the shortest release site-to-trap distance. Second, suspension of trap operations during peak hatchery releases omitted the data which could lend the most support for testing the pied piper hypothesis.

Previous studies reported that temperature experience [47], stream temperatures [31], and temperature residuals [25] strongly influenced the migration of Chinook salmon. Temperature difference had the strongest (negative) effect on the number of wild Chinook salmon in the Dungeness River. Thus a drop in water temperature seemed to cause Chinook salmon to migrate, as can occur when rivers rise with melting snow after an increase in air temperatures or rain on snow events [52], and increased flows in this river were correlated with more Chinook migrating. In line with this expectation, in the Puyallup River, air temperature anomalies had a positive effect on the number of Chinook salmon migrating. The correlation between a drop in water temperature and the increase in the number of salmon smolts migrating, previously reported in coho salmon [48], was contrary to our expectation since warmer temperatures resulted in earlier migration in Chinook salmon [47]. We found that flow difference had a strong effect on both Chinook and coho salmon in the Dungeness and Puyallup rivers. However, flow difference was not a covariate in the best model for either species in the Skagit River. Flow anomaly had a small negative effect on all coho populations. Perhaps flow is more important in smaller rivers like the Dungeness and Puyallup where smaller changes in flow can have larger effects on the fish. Flow might also be less important in the Skagit River because it has several dams that affect flows, whereas the Dungeness River does not currently have any dams. The Puyallup River upstream of the smolt trap has one dam, and its floodplain habitat is more degraded than the other rivers. Its flow varies widely, and so an increase in the flow can provide a cue for migration.

The three rivers examined here are not replicates of each other; they differ in many abiotic and biotic attributes. The salmon populations have adapted to these conditions, so the fish are not replicates of each other either [53, 54]. Furthermore, different species and populations of Pacific salmon respond differently to changing environmental conditions, not all populations will alter migratory behaviors in unison with one another [55]. Consequently, the varied effects of different abiotic factors (temperature, flow, etc.) on migrating salmon in the species-river combinations are not unexpected. Similarly, the abiotic and social effect results were not all entirely consistent. We interpret this as indicating the complexity of factors involved in initiating migration, rather than evidence against the effect of any specific factor. The three rivers also differed in the distances between the hatchery release site and the trap. The distance between the release site and the smolt trap determines the ‘fetch’ over which a wave of hatchery smolts can accumulate wild smolts and therefore sets the potential extent of social influence from hatchery fish that can be detected at the trap. Moreover, the nature of the social effect cannot be discerned from correlational studies such as this. That is, we envision that the wild salmon are stimulated to join the downstream migrating hatchery fish and so are “swept along” with them but it is also possible that in some manner the wild fish are pushed downstream by some form of agonistic interaction. Given the general breakdown of aggressive behavior among smolts and previous visual observation of wild smolts being “pulled” downstream by hatchery smolts [39], this seems much less likely than that they are being “pushed,” but the “push” hypothesis cannot be excluded. Regardless of the mechanism, the detection of correlated movements consistent with social effects on migration in the context of environmental variation is our salient finding. Of course, this is a correlational study, not an experimental one, so alternative explanations remain possible.

Although the data from salmon traps are highly appropriate for the purposes of our study and collected “blind” with respect to our hypotheses, there remain some limitations to our data that warrant consideration. First, analyses assume that all unmarked individuals are ‘wild’, which we explored by looking at the correlation between the number of unmarked hatchery salmon released and the number of wild salmon caught in the traps (Figure S2). We found only a low-to-moderate correlation between the two, implying that the increase in the number of wild salmon was not exclusively because of the unmarked hatchery salmon. Where a correlation did exist, our sensitivity analysis suggested that the results were not due to unmarked hatchery fish (Figure S20, S21, S22). Second, the time scale at which data were collected at the traps might not be the time scale of the social influence we tried to study. If wild salmon respond to the hatchery salmon immediately or within a few hours, the effect of this social influence would be diluted by recording data only every 12 h. Due to this potential mismatch, we suspect our results underestimate the actual social effect. Third, as noted above, we could not determine the mechanism of the effect; were the wild smolts led by hatchery fish (pied piper hypothesis) or displaced by them? Fourth, although we attempted to account for common environmental cues, we cannot exclude the possibility that both hatchery and wild fish responded to stimuli external to our model, rather than hatchery fish stimulating wild fish to migrate. Indeed, hatchery managers often deliberately release fish to match wild migration timing, so some level of hatchery-wild correlation was expected. Follow up experiments, either in a simulated stream setting, or monitoring wild fish behavior following deliberate manipulation of hatchery releases, would help reduce the uncertainty associated with the correlational approach presented here. Finally, the suspension of trap operations following a hatchery release resulted in the loss of data most germane to understanding the influence of hatchery smolts on their wild counterparts.

Despite the limitations of the dataset and the potential dilution of the effect caused by missing data and the time resolution of the data, our results suggest that along with environmental covariates, social information provided cues for salmon seaward migration. Thus future studies could consider social cues from hatchery and wild conspecifics as well as environmental effects on migration timing of salmonid smolts. If large hatchery releases motivate the wild salmonid smolts to migrate with them, this can have fundamental ecological consequences. Wild smolts that are being influenced by hatchery smolts might migrate earlier than they normally would, encounter less than optimal foraging conditions and could encounter more predators, thereby experiencing higher mortality at sea [25]. As a result of early migration, juvenile wild salmonids may enter the estuary less ready for saltwater, which can extend residence time and thereby increase exposure to predators [56]. On the other hand, wild salmonids migrating alongside hatchery fish might benefit from predator swamping [57].

The evidence that salmon smolts use social information provides perspectives on their behavior in the pre-industrial period as well as implications for the integration of wild and hatchery populations. Strong density-dependence in the freshwater stages is typical of Chinook [44] and coho salmon [58] and steelhead [59]. Consequently, the disparity in numbers of returning adults in past centuries compared to current levels might have been much greater than for smolts. In the absence of hatchery production, wild smolts would still have responded to the pulses of other smolts, though the overall effect might have been more muted compared to the effects of large pulses of hatchery fish. These pulses might have altered the timing of migration for some individuals, for better or worse, but also conferred survival benefit from predator swamping (e.g. [60, 61]).

Salmon hatcheries were set up in response to overfishing, habitat loss, and other factors decreasing salmon runs, and they provide many sociocultural and economic benefits. Hatcheries are operated to increase abundance for fishing or promote conservation of imperiled populations, but they can have unintended ecological consequences for wild populations [40, 62, 63]. Our results suggest that hatcheries could experiment with different release strategies to reduce effects on wild populations. For example, hatcheries can consider timing their releases towards the end of the migration period of the wild fish or consider releasing the hatchery fish downstream of the wild fish habitat. Some hatcheries are moving to volitional releases, which may spread the migration of hatchery smolts over a longer period of time [64]. This practice may reduce the pied piper effect, if the wild smolts respond to a high threshold number of hatchery smolts, or may increase the occurrence of the pied piper effect, by providing a longer social cue to influence the wild smolts. Monitoring changes in wild smolt migration timing patterns following experimental release practices would provide valuable information on the frequency and magnitude of hatchery social effects.

Our findings, indicating social effects on Pacific salmon smolt migration timing, fit two nascent patterns in the field of collective behavior. First, our results support an increasing recognition of the importance of sociality in salmon, from navigation [65, 66] to trophic interactions [67]. Second, our results expand a growing body of literature on the importance of social information on timing decisions in diverse taxa [11, 35, 68–71]. It remains to be seen if, and how, collective timing decisions may improve the accuracy of those decisions [12]. If salmon, and other species, do improve decision making with social information, especially during fitness-critical events such as migration, reductions in abundance could decrease decision making ability and accelerate population declines [72–74]. We hope our results encourage more studies exploring social influence on timing decisions and the potential population-level implications.

## Supplementary Information

Below is the link to the electronic supplementary material.

Supplementary Material 1

## Data Availability

The smolt trap data analyzed for this study are owned by the Puyallup Tribe of Indians (PTI) and the Washington Department of Fish & Wildlife (WDFW) and are not publicly available, however the data are available from PTI and WDFW on reasonable request. All the code used to process and analyze the data are available on github: [maria-kuruvilla/pied\_piper\_marss\_final: Final analysis to test pied piper hypothesis.] (https://github.com/maria-kuruvilla/pied_piper_marss_final).
