# Supplementary material for: Social influences complement environmental cues to stimulate migrating juvenile salmon
Source: Mov Ecol. 2026 Apr 6;14:33. doi: 10.1186/s40462-026-00644-y (PMC13188761; doi:10.1186/s40462-026-00644-y)
Supplement: Supplementary file 1 — Supplementary Material 1 [file 40462_2026_644_MOESM1_ESM.docx]

## Supplementary Information for: Social influences complement environmental cues to stimulate migrating juvenile salmon
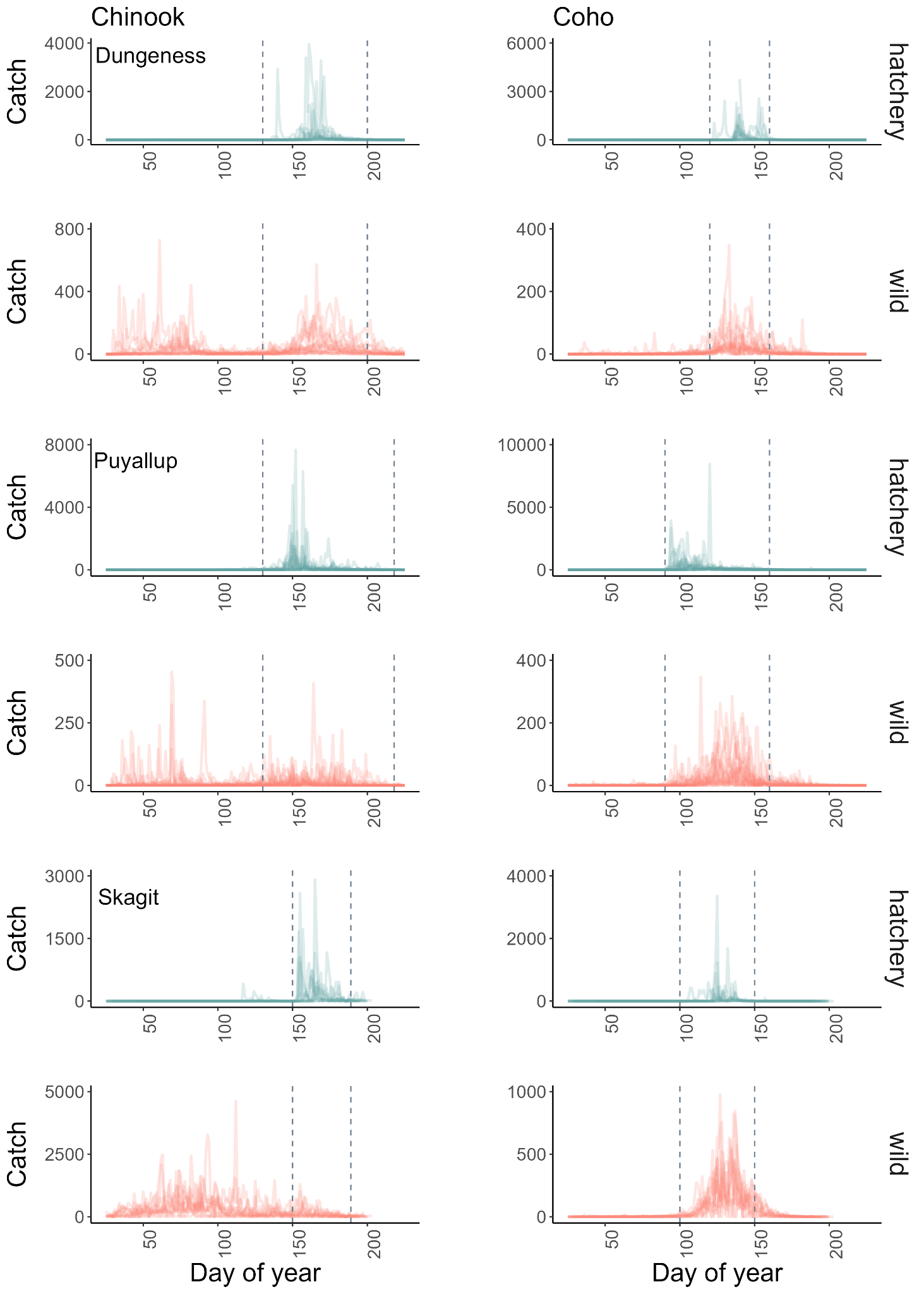


Figure S1 - Daily catches for hatchery and wild sub-yearling Chinook salmon, hatchery and wild yearling coho salmon in the Dungeness (years 2005-2020), Puyallup (years 2004-2021), and Skagit rivers (years 2010-2022). Raw data without interpolation from every year available is overlaid. The vertical dashed lines bracket the period when both hatchery and wild salmon were caught in the trap, which was the portion of data we used for the analysis.

| River | Species | Origin | Total number caught | Max number caught |
| --- | --- | --- | --- | --- |
| Dungeness | Chinook | hatchery | 62146 | 3274 |
| Dungeness | Chinook | wild | 42836 | 355 |
| Dungeness | Coho | hatchery | 58857 | 1983 |
| Dungeness | Coho | wild | 15414 | 348 |
| Puyallup | Chinook | hatchery | 153030 | 7429 |
| Puyallup | Chinook | wild | 15360 | 342 |
| Puyallup | Coho | hatchery | 155593 | 8436 |
| Puyallup | Coho | wild | 27759 | 291 |
| Skagit | Chinook | hatchery | 51332 | 2912 |
| Skagit | Chinook | wild | 47285 | 982 |
| Skagit | Coho | hatchery | 29207 | 3364 |
| Skagit | Coho | wild | 83347 | 976 |

Table S1 - Total and maximum number of fish caught in the trap during one session and counted by a trap operator during the days and years used in our analysis.

### Unmarked hatchery salmon


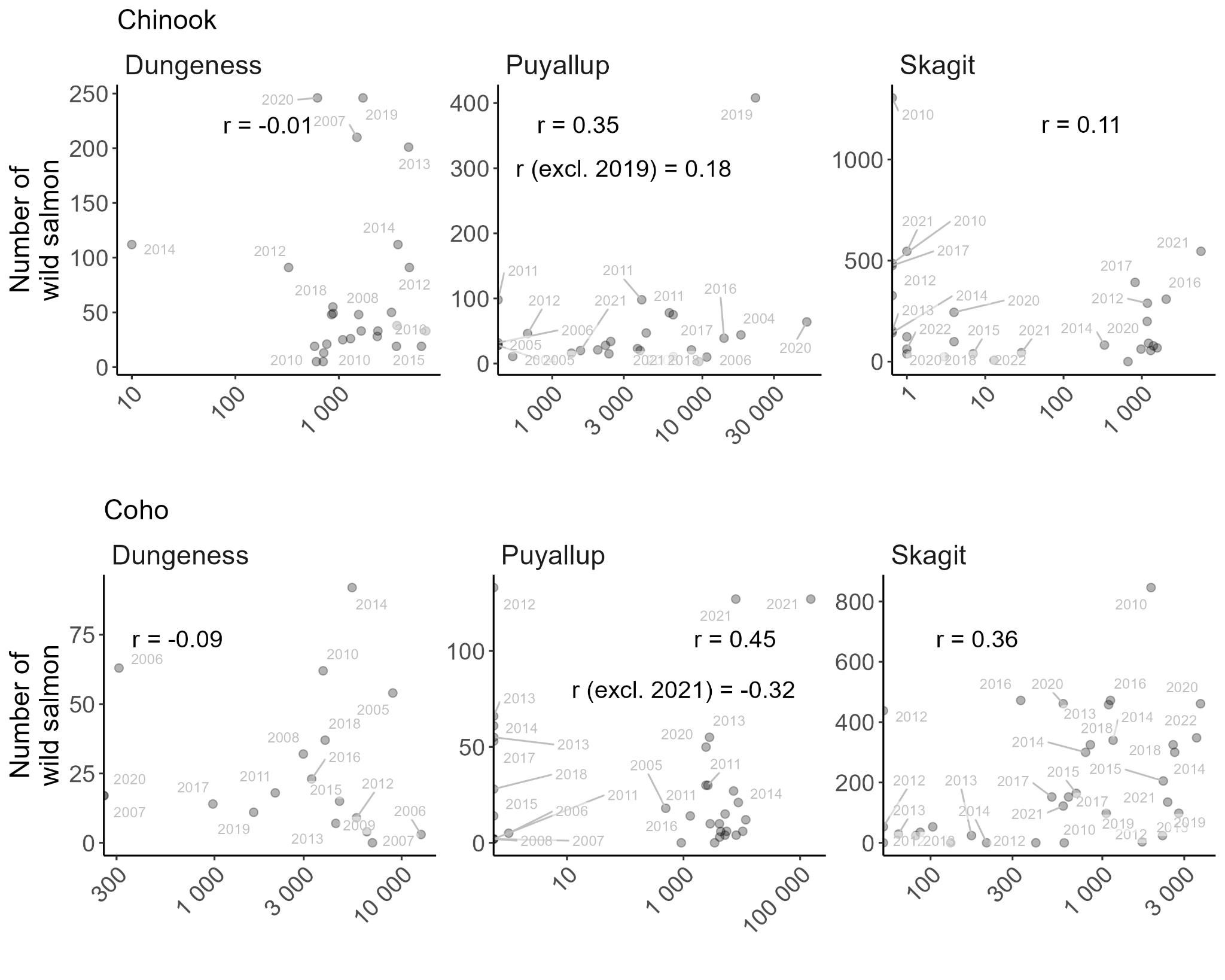


Figure S2 - Pearson correlation coefficient between unmarked hatchery salmon released by hatchery and wild salmon subsequently caught in the trap for Chinook and coho salmon in the Dungeness, Puyallup, and Skagit rivers. Every hatchery release has the number of unmarked hatchery salmon associated with it, which is estimated by the hatchery and reported on the RMIS website. In order to match a hatchery release with the wild salmon caught in the trap, we identified, for each release, the day within the following 10 days on which the number of hatchery salmon caught was maximal. Each data point corresponds to one of these peak-catch days. The x-axis is shown on a logarithmic scale for visualization only.

### Proportion of day vs. night migrants


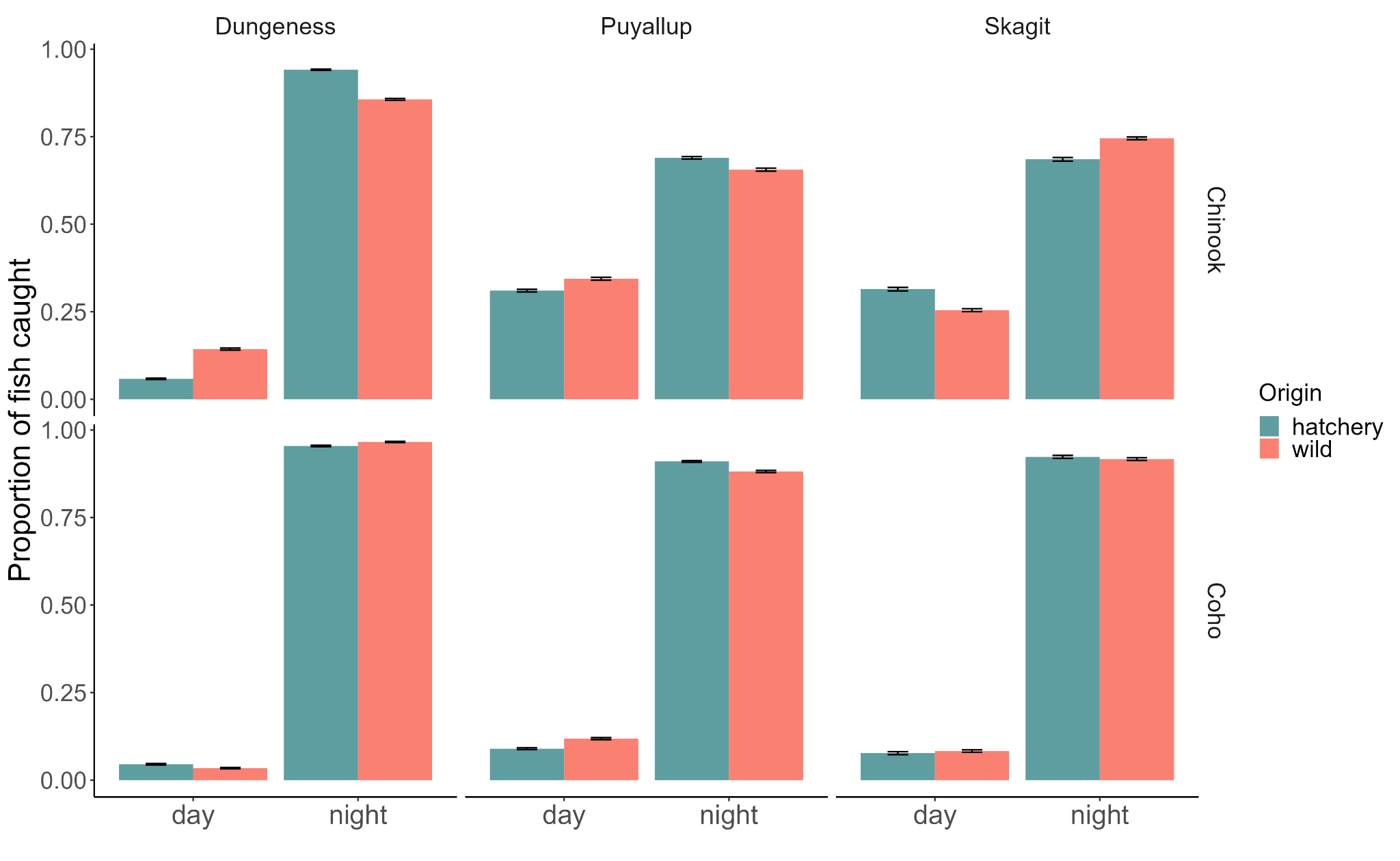


Figure S3 - Average proportion of Chinook salmon and coho salmon caught in the trap at night vs. day in all three rivers.

### Temperature and Flow data


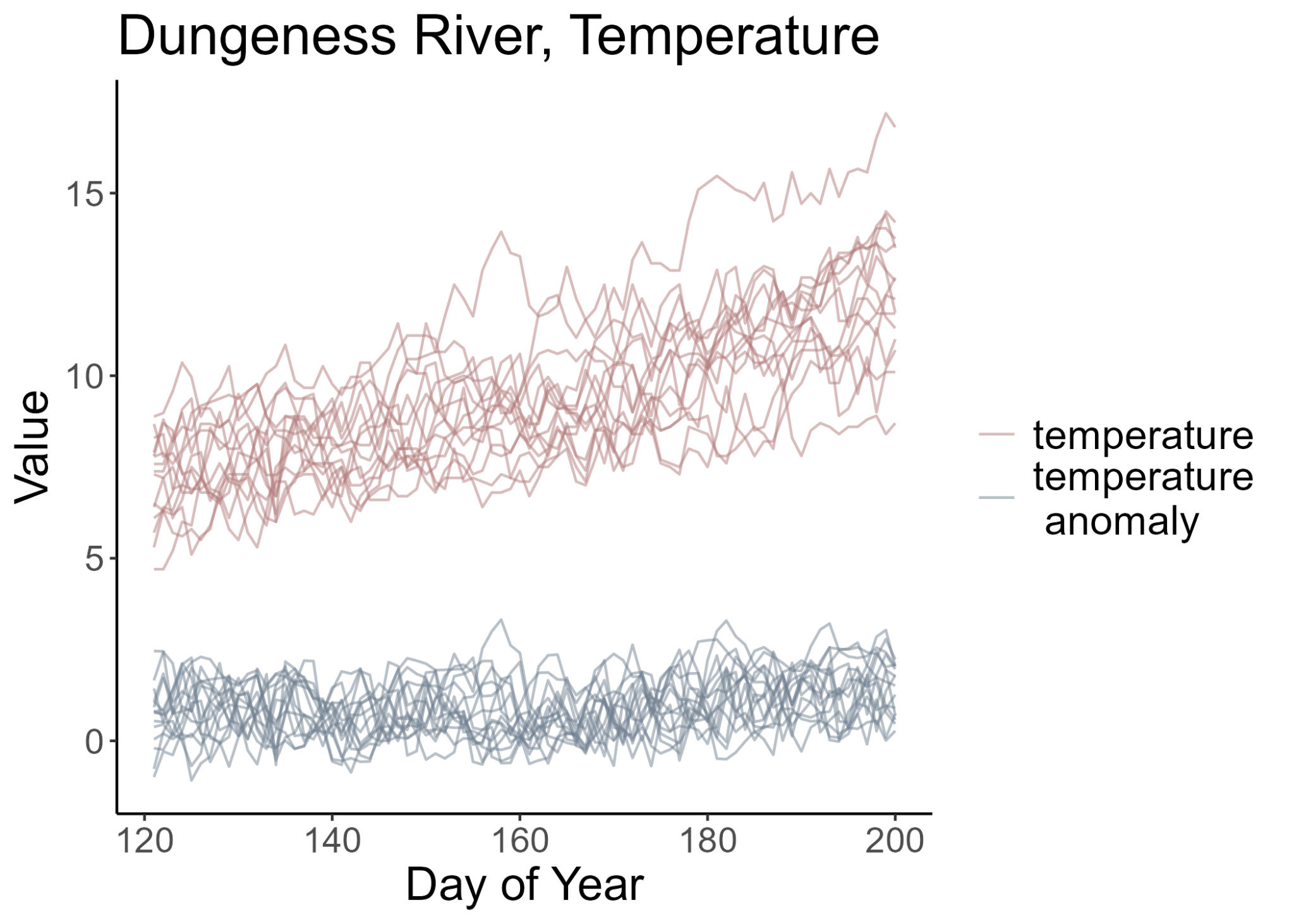

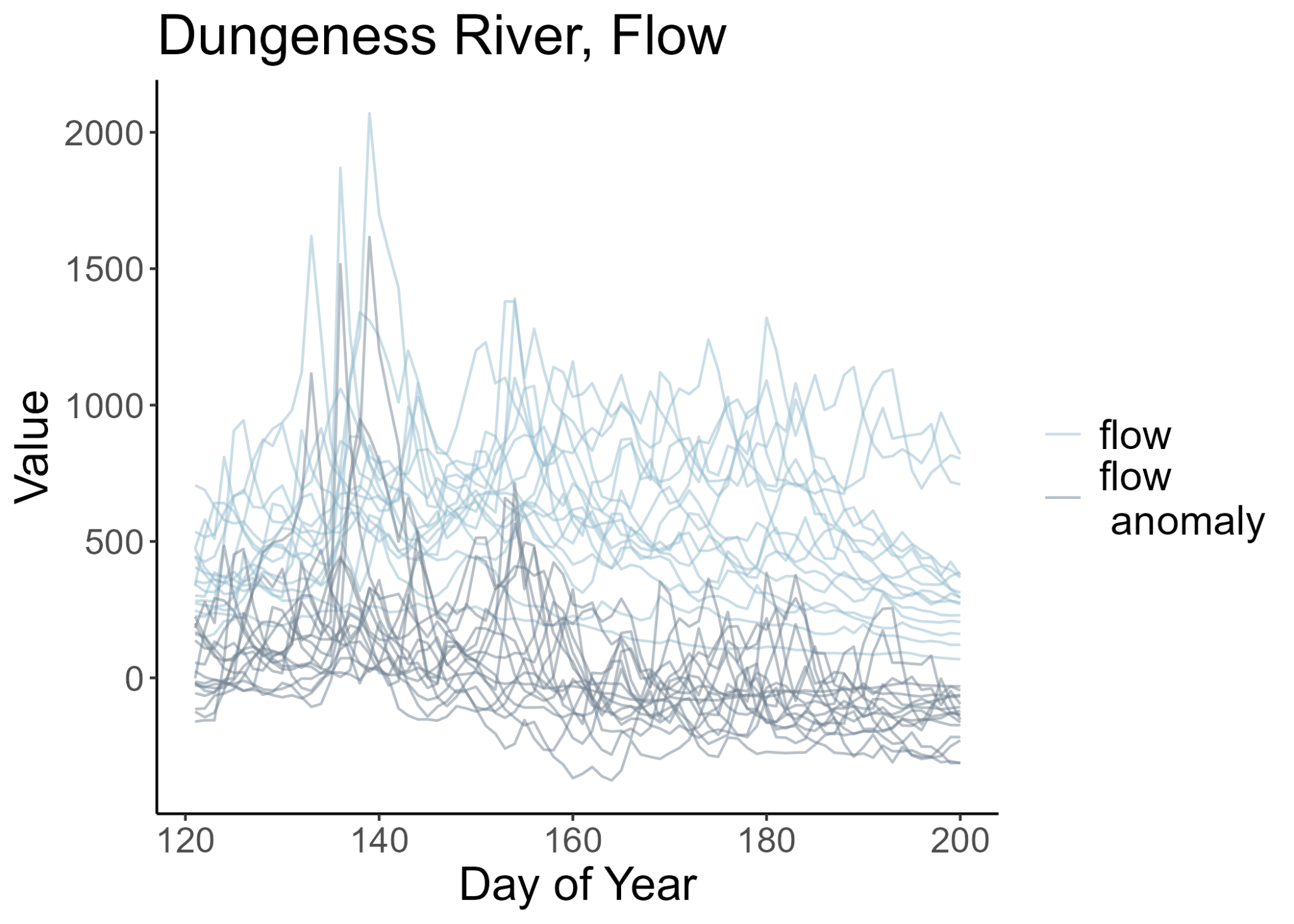


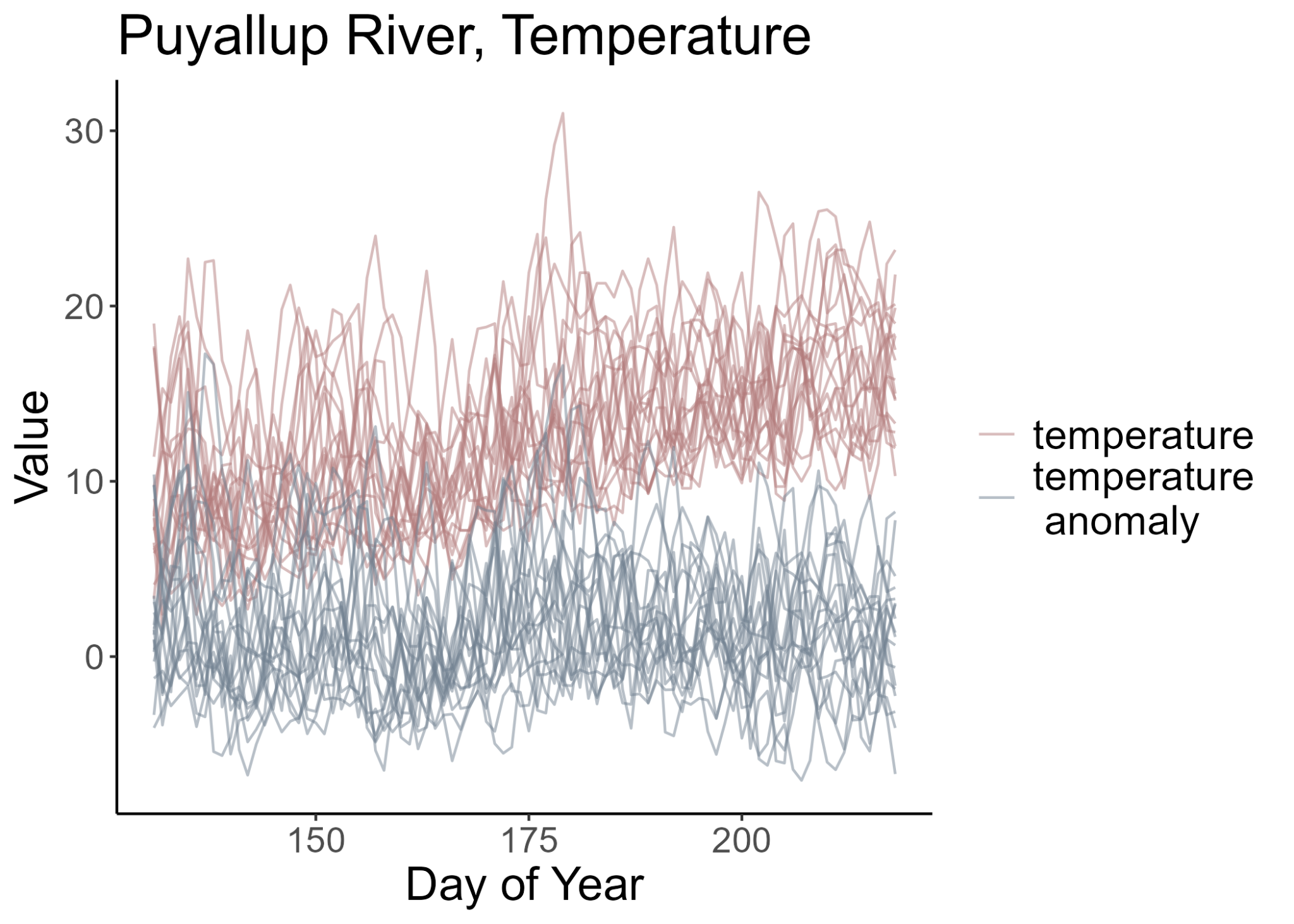

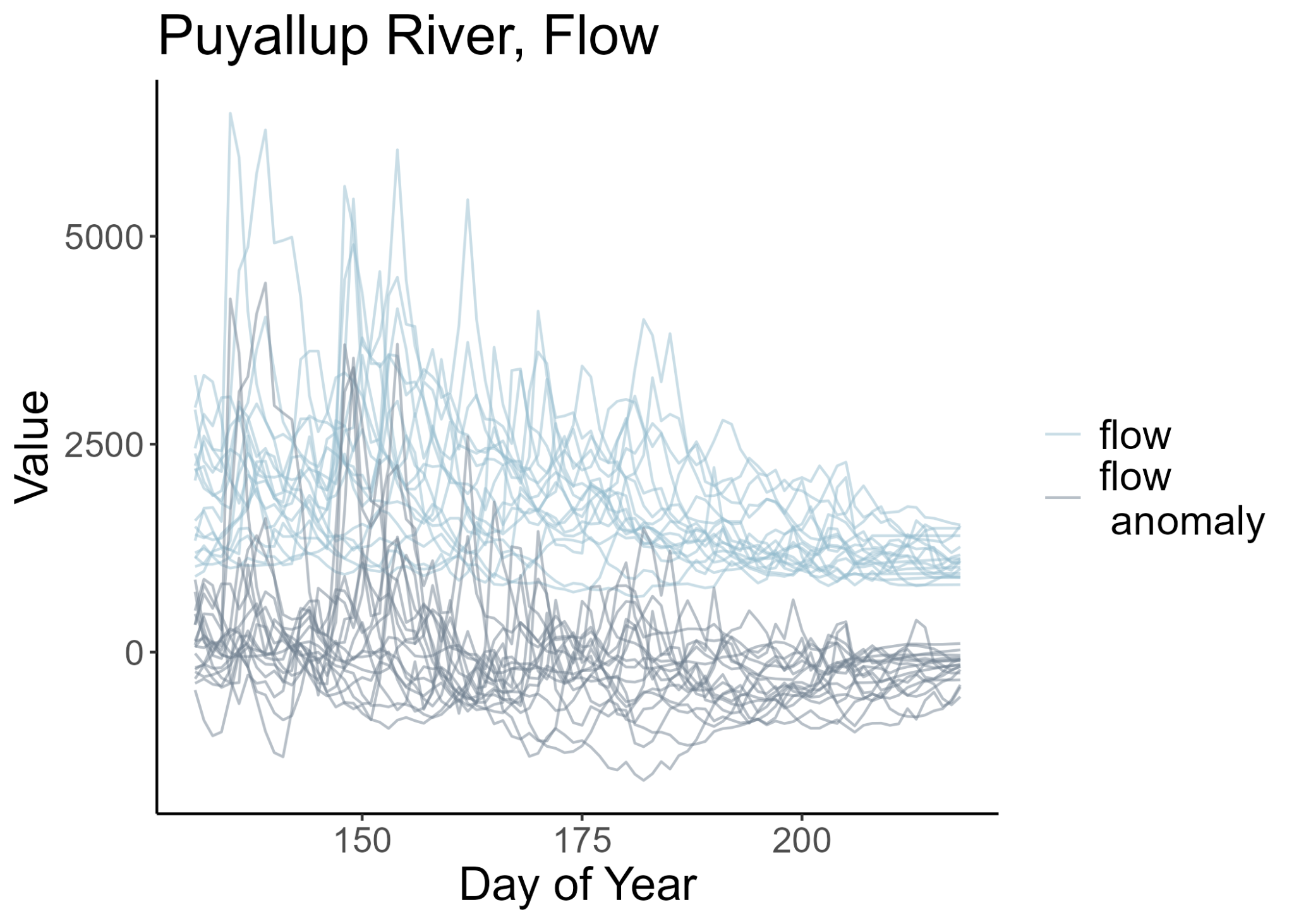


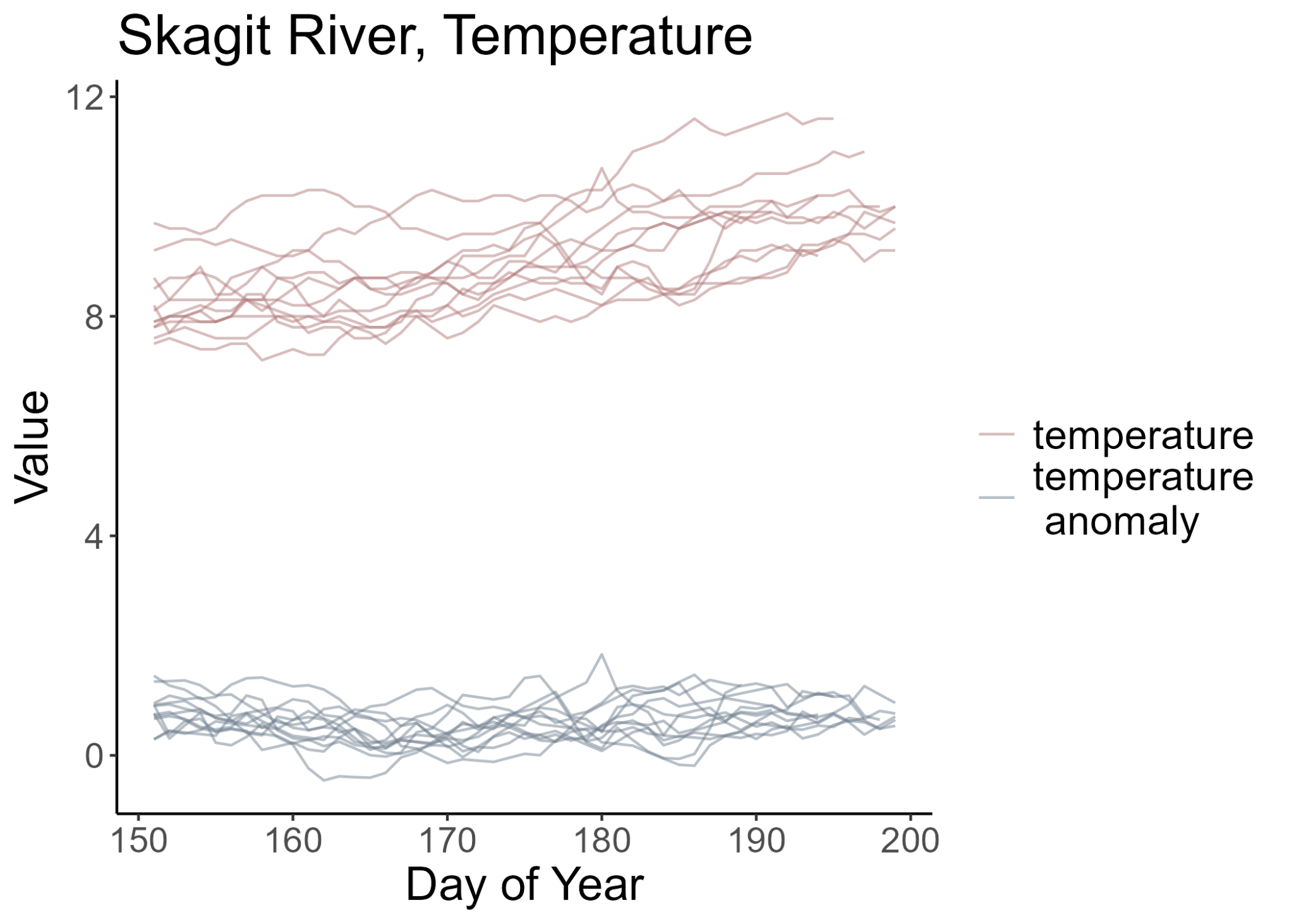

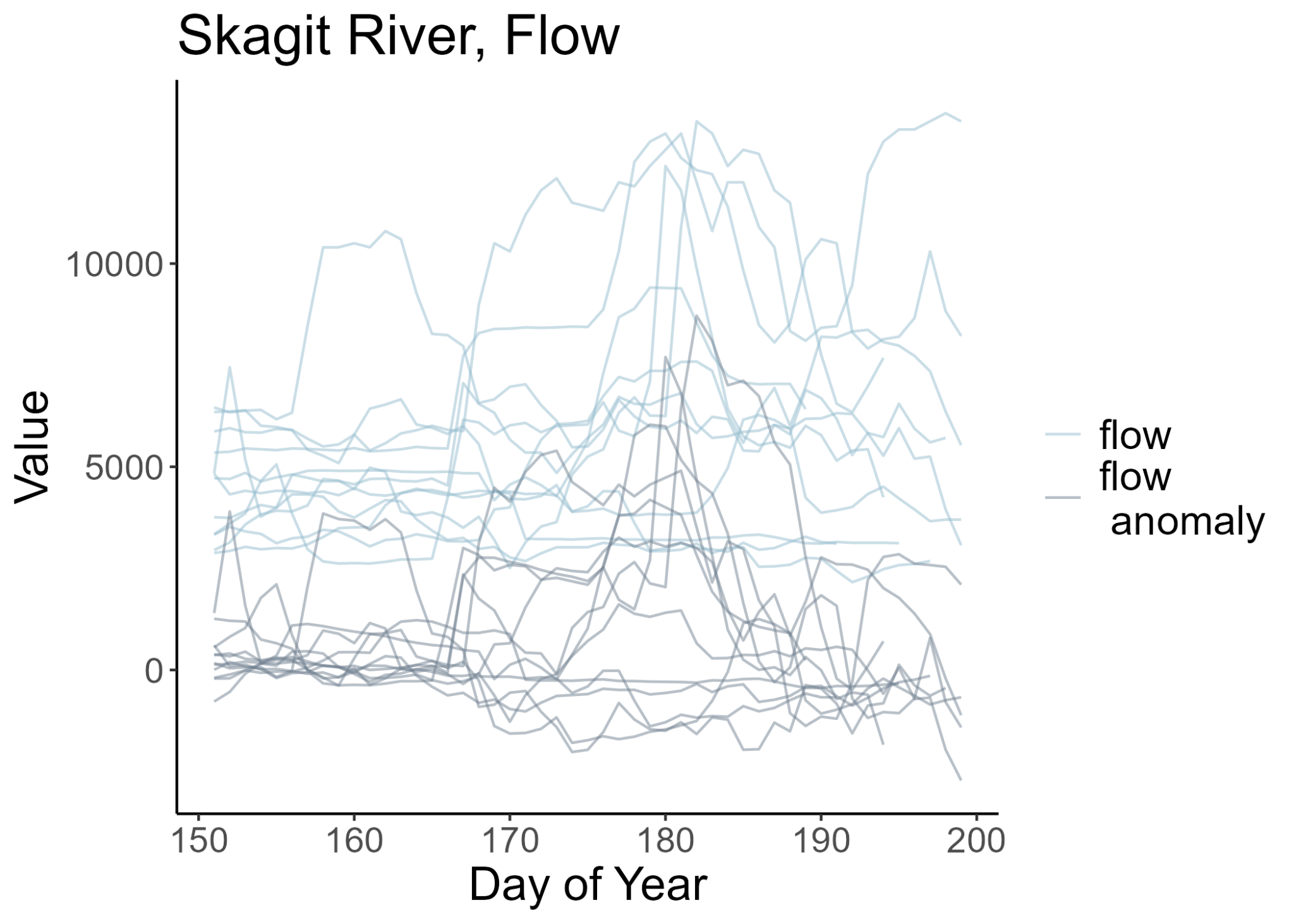


Figure S4 - Temperature and Flow data and anomalies for the Dungeness, Puyallup, and Skagit rivers.

### Dungeness River

The Dungeness River is a 45-km river in the northwest part of Washington State. It originates in the Olympic Mountains and drains into the Strait of Juan de Fuca. The Dungeness River is fed through melting snowpack from the Olympic Mountains and by rain. The lowest flows are in August-September whereas the highest temperature is around 15° C in July-August. The Dungeness River supports seven species of salmonids - Chinook, coho, chum and pink salmon, steelhead, cutthroat and bull trout. There are multiple hatcheries that release Chinook and coho salmon and steelhead into the river at various stages. The Dungeness River hatchery, Gray Wolf, Hurd Creek, and Upper Dungeness Hatchery are the main hatcheries on the Dungeness River. A rotary screw trap is used to monitor juvenile salmon that migrate from their natal site to the ocean. The trap, operated by the Washington Department of Fish and Wildlife (WDFW), is located about 1.6 km upstream of the Strait of Juan De Fuca (48.1441, -123.1283).


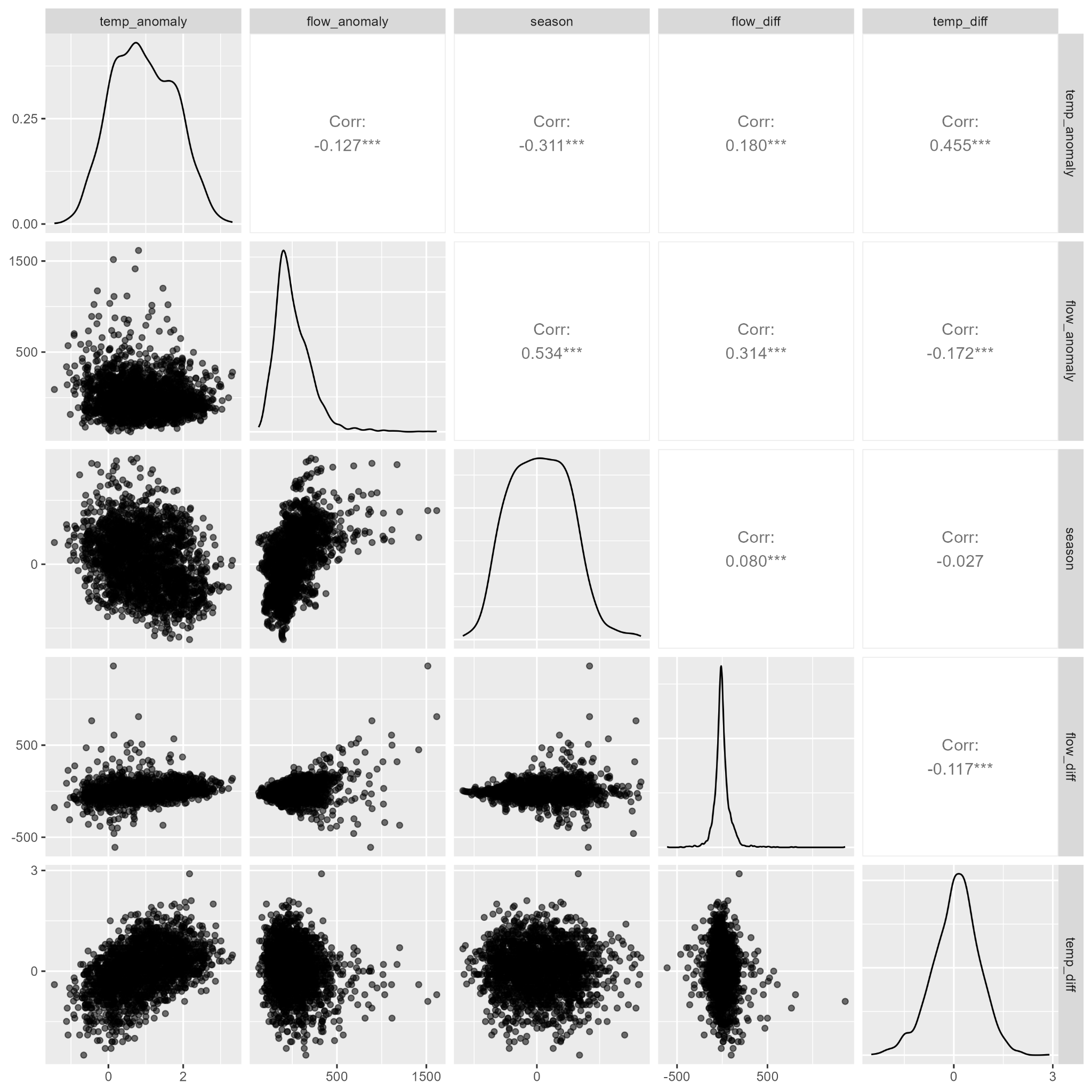

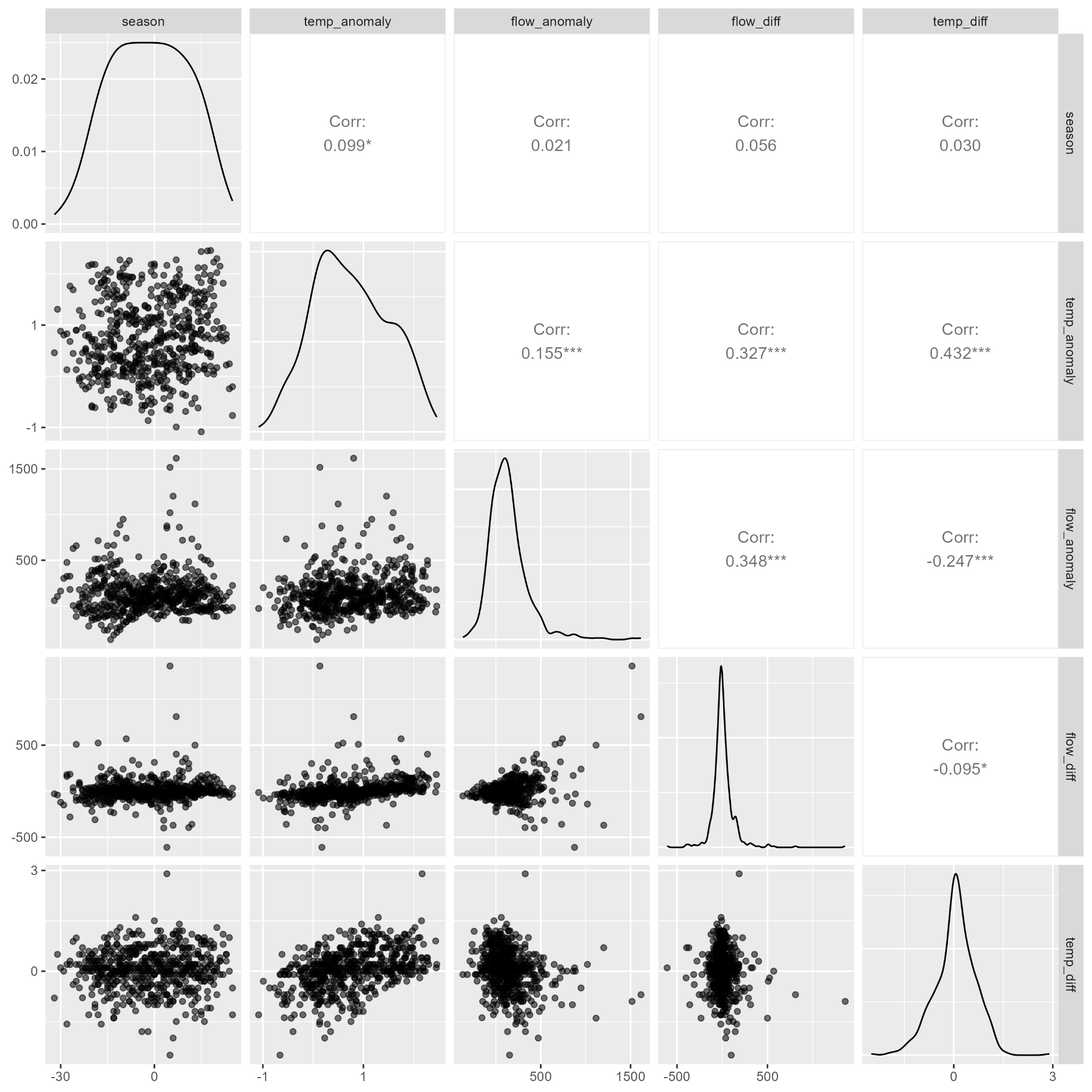


Figure S5 - Correlation between all the environmental covariates in the Dungeness River for the day of year 130-200 included in the Chinook model (top) and for day of year 120-160 included in the coho model(bottom). The asterisks denote significance. The diagonal shows the probability density functions for each variable.

| **Chinook** | | **Coho** | |
| --- | --- | --- | --- |
| **Error structure** | $\Delta AICc$ | **Error structure** | $\Delta AICc$ |
| Equal | 18.5 | NA | NA |
| Unequal | 0 | NA | NA |

Table S2 - Model selection with different error structure for day and night for Chinook salmon and coho salmon in the Dungeness River.

| **Chinook** | |  | |
| --- | --- | --- | --- |
| **Covariate** | $\Delta AICc$ |  |  |
| temperature difference | 0 |  |  |
| season | 39.6 |  |  |
| flow anomaly | 40.0 |  |  |
| temperature anomaly | 70.1 |  |  |

Table S3 - Model selection for Chinook salmon in the Dungeness with each of the correlated covariates.

| Temperature anomaly | Season | Flow difference | Temperature difference | Hatchery, day | Hatchery, night | $\Delta AICc$ |
| --- | --- | --- | --- | --- | --- | --- |
| NA | 0.03 | 0.06 | -0.14 | 0.24 | 0.15 | 0 |
| 0 | 0.04 | 0.06 | -0.14 | 0.24 | 0.15 | 1.96 |
| 0.02 | 0.04 | NA | -0.14 | 0.25 | 0.15 | 14.94 |
| NA | 0.04 | NA | -0.13 | 0.25 | 0.15 | 16.18 |
| -0.02 | NA | 0.08 | -0.13 | 0.24 | 0.15 | 20.11 |
| NA | NA | 0.08 | -0.14 | 0.24 | 0.15 | 20.55 |
| NA | NA | NA | -0.14 | 0.25 | 0.16 | 45.27 |
| 0 | NA | NA | -0.14 | 0.25 | 0.16 | 47.34 |
| -0.02 | 0.03 | 0.07 | NA | 0.25 | 0.15 | 64.7 |
| NA | 0.04 | 0.06 | NA | 0.26 | 0.15 | 67.31 |

Table S4 - Top ten models from model selection with all combinations of uncorrelated covariates for Chinook subyearlings in the Dungeness River.

| **Chinook** | | **Coho** | |
| --- | --- | --- | --- |
| **Variable** | **Relative Importance** | **Variable** | **Relative Importance** |
| Temperature difference | 1 | Season | 1 |
| Flow difference | 1 | Flow difference | 1 |
| Season | 1 | Flow anomaly | 0.98 |
| Hatchery difference | 1 | Hatchery difference | 0.4 |
| Temperature anomaly | 0.27 | Temperature anomaly | 0.32 |
|  |  | Temperature difference | 0.26 |

Table S5 - Relative variable importance for all variables used in the model selection process for sub-yearling Chinook salmon and yearling coho salmon in the Dungeness River.

| Season | Temperature anomaly | Flow anomaly | Flow difference | Temperature difference | Hatchery difference | $\Delta AICc$ |
| --- | --- | --- | --- | --- | --- | --- |
| 0.09 | NA | -0.03 | 0.07 | NA | NA | 0 |
| 0.09 | NA | -0.03 | 0.07 | NA | 0.03 | 0.82 |
| 0.09 | 0.01 | -0.03 | 0.06 | NA | NA | 1.51 |
| 0.09 | NA | -0.03 | 0.07 | -0.01 | NA | 2.11 |
| 0.09 | 0.01 | -0.03 | 0.06 | NA | 0.03 | 2.28 |
| 0.09 | NA | -0.03 | 0.07 | -0.01 | 0.03 | 2.92 |
| 0.09 | 0.01 | -0.03 | 0.07 | -0.01 | NA | 3.6 |
| 0.09 | 0.01 | -0.03 | 0.06 | -0.01 | 0.03 | 4.36 |
| 0.09 | NA | NA | 0.04 | NA | NA | 7.07 |
| 0.09 | NA | NA | 0.04 | NA | 0.02 | 8.81 |

Table S6 - Estimates of the covariates from the top ten models in the model selection with all combinations of uncorrelated covariates for coho salmon in the Dungeness River.


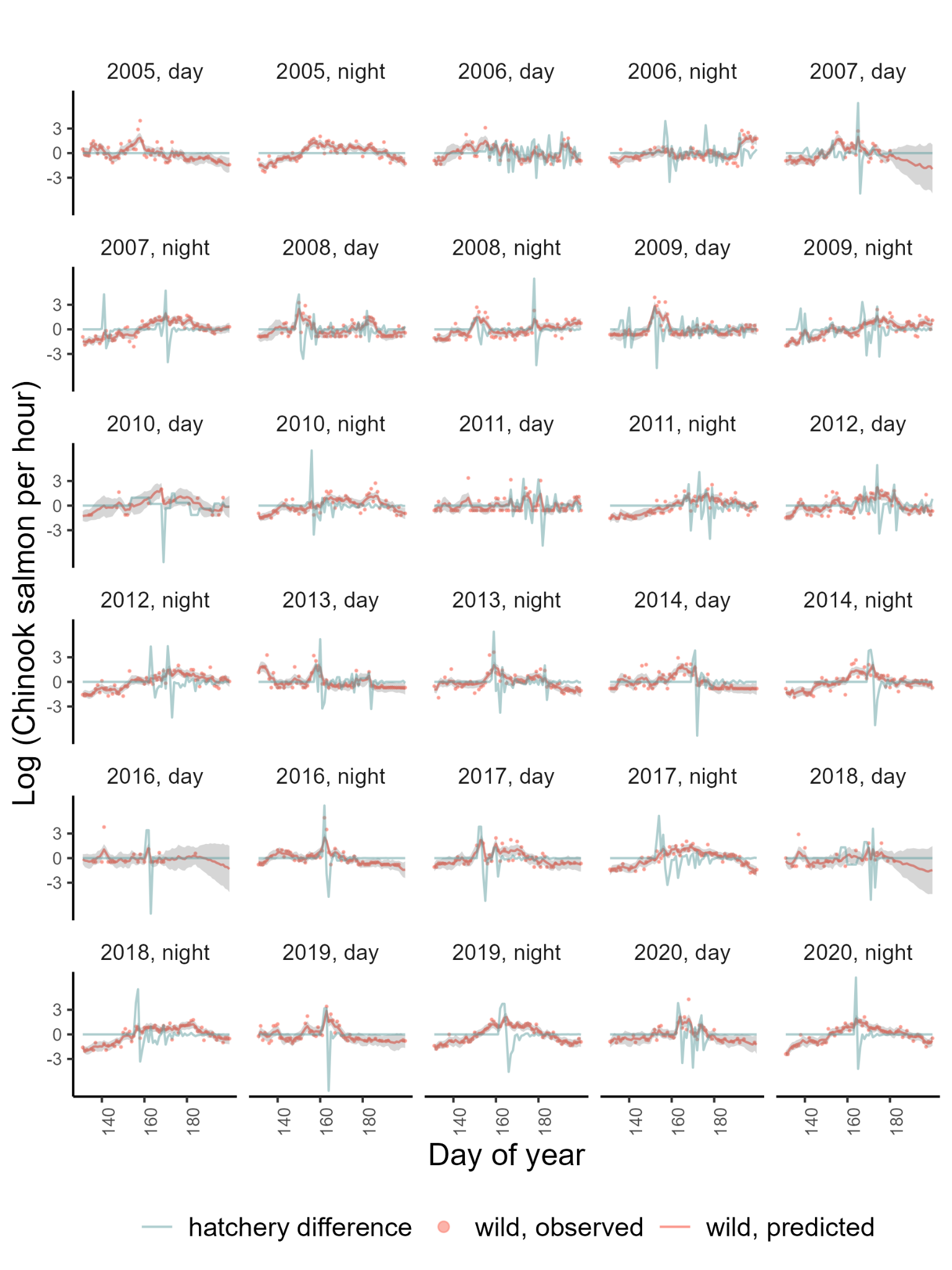


Figure S6 - Model estimates and observations of wild Chinook salmon and observations of hatchery Chinook salmon in the Dungeness River.


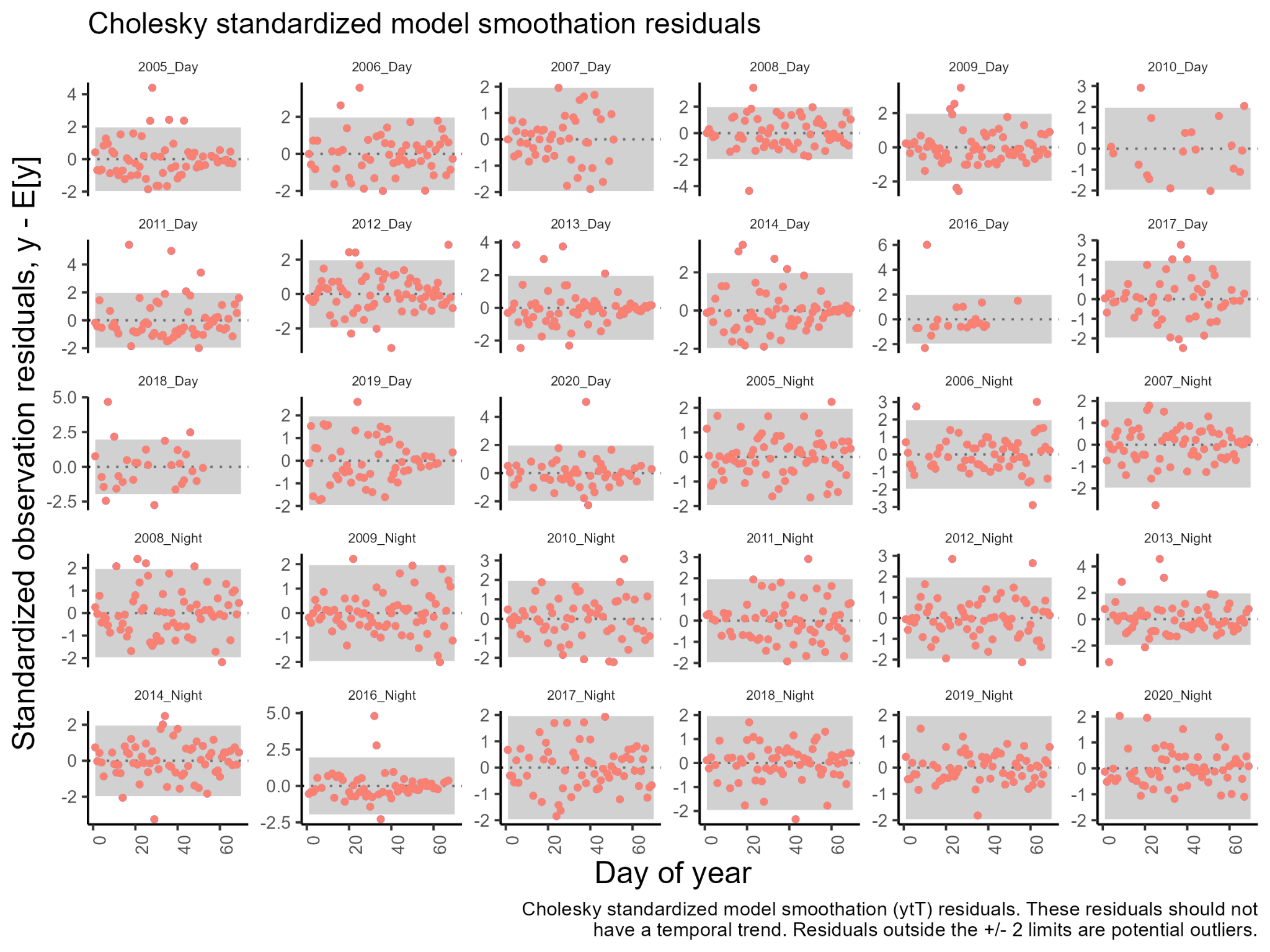


Figure S7 - Model residuals from the best model for Chinook salmon in the Dungeness River.


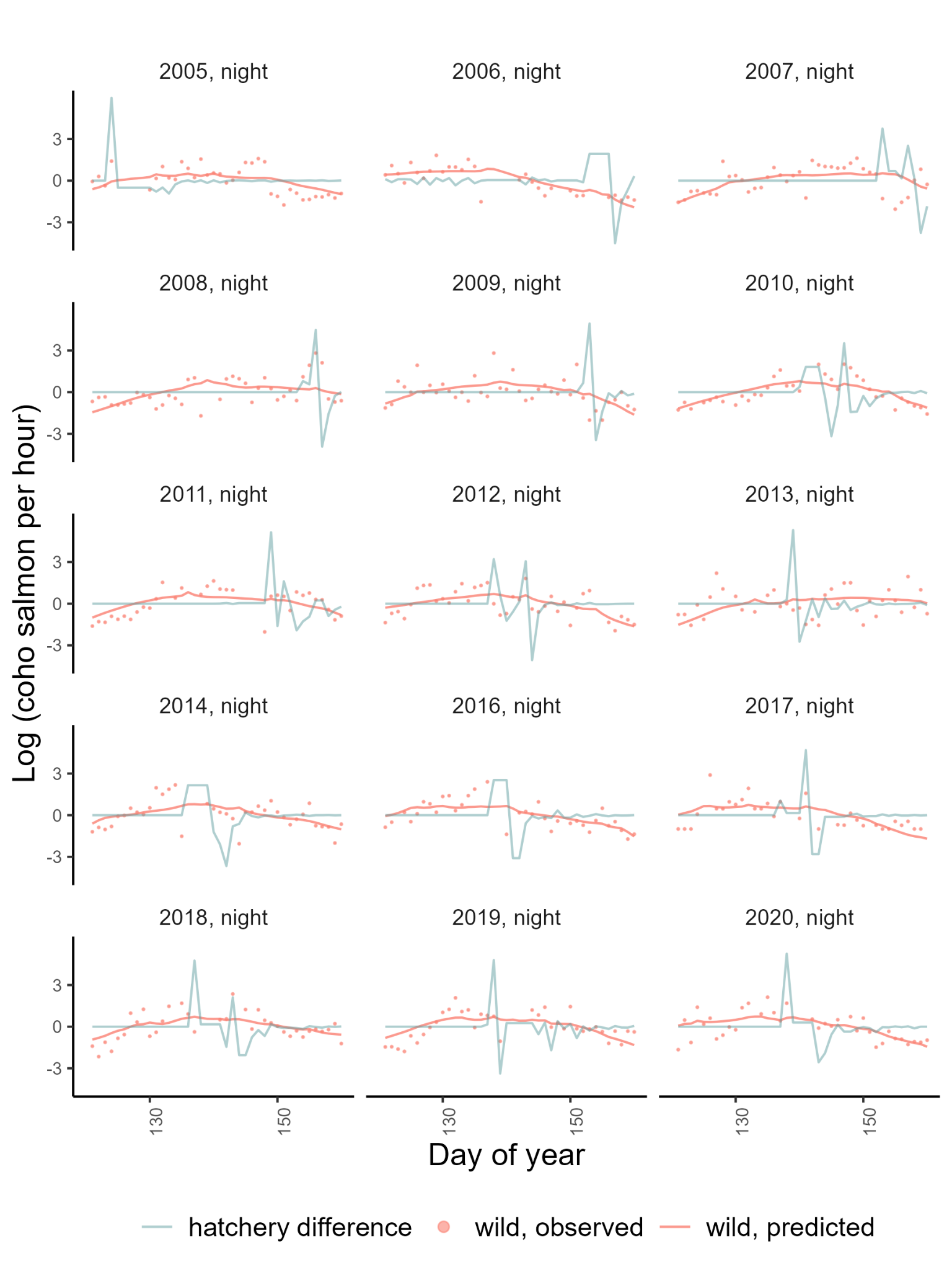


Figure S8 - Model estimates and observations of wild coho salmon and observations of hatchery coho salmon in the Dungeness River


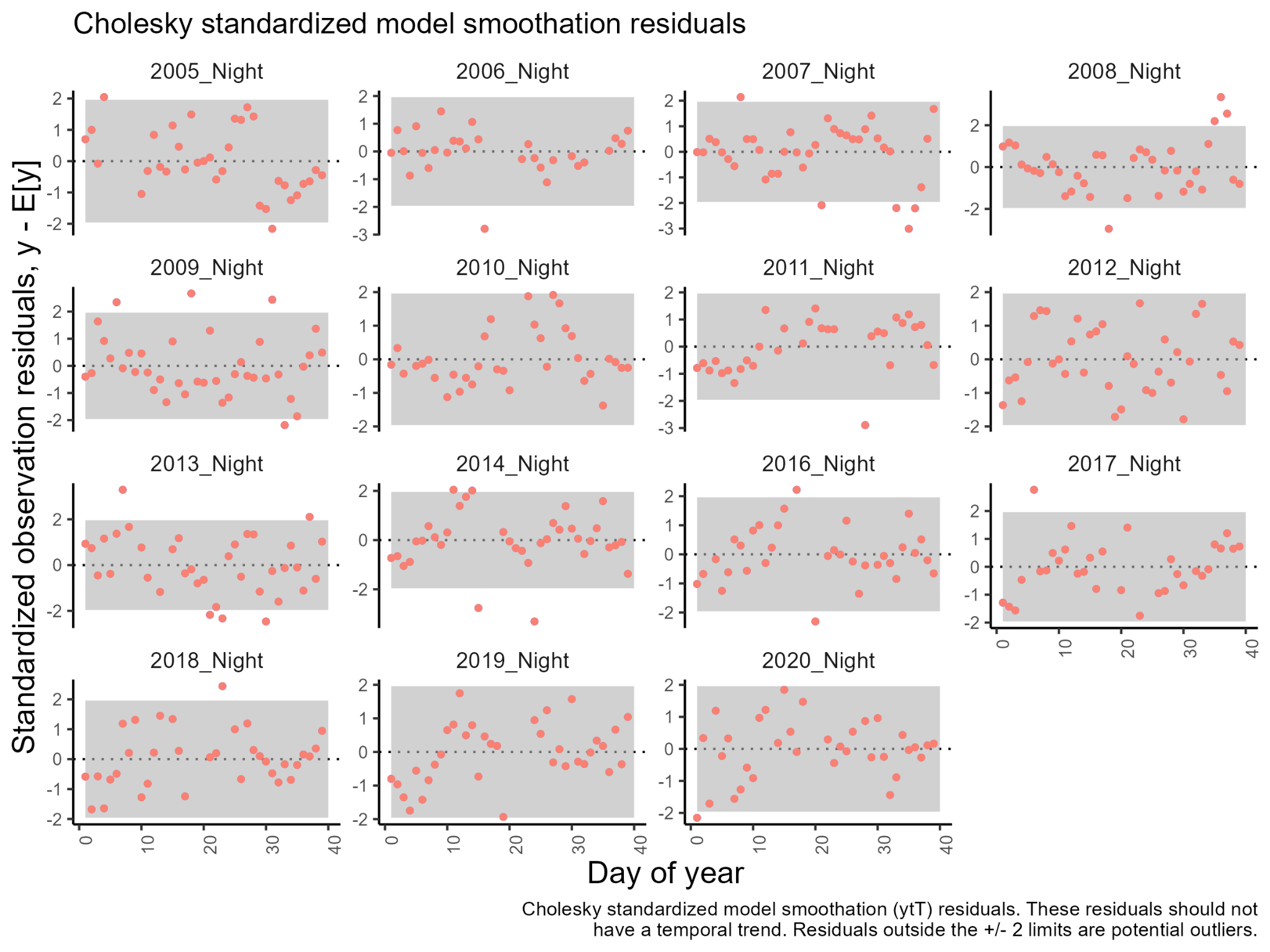


Figure S9 - Model residuals from the best model for coho salmon in the Dungeness River.

### Puyallup River

The Puyallup River is a 72-km river that originates in the glaciers on Mount Rainier and drains into Puget Sound. In the summer, the river is fed by melting glaciers which increases the turbidity. River flow and sediment budget is largely natural, except a diversion dam located in the upper Puyallup which diverts and returns a portion of the river for hydropower. The highest water temperatures are around July-August and the lowest flows are in September-October. The Puyallup River supports Chinook, coho, pink, and chum salmon, and steelhead, cutthroat and bull trout. The Puyallup River trap, operated by the Puyallup Tribe of Indians, is located just upstream of the confluence of the White River and Puyallup River (47.1971, -122.2523). The trap catches hatchery salmon from the Voights Creek hatchery, and Rushingwater, Cowskull, and Wilkeson Creek acclimation ponds and Lake Kapowsin net pen.


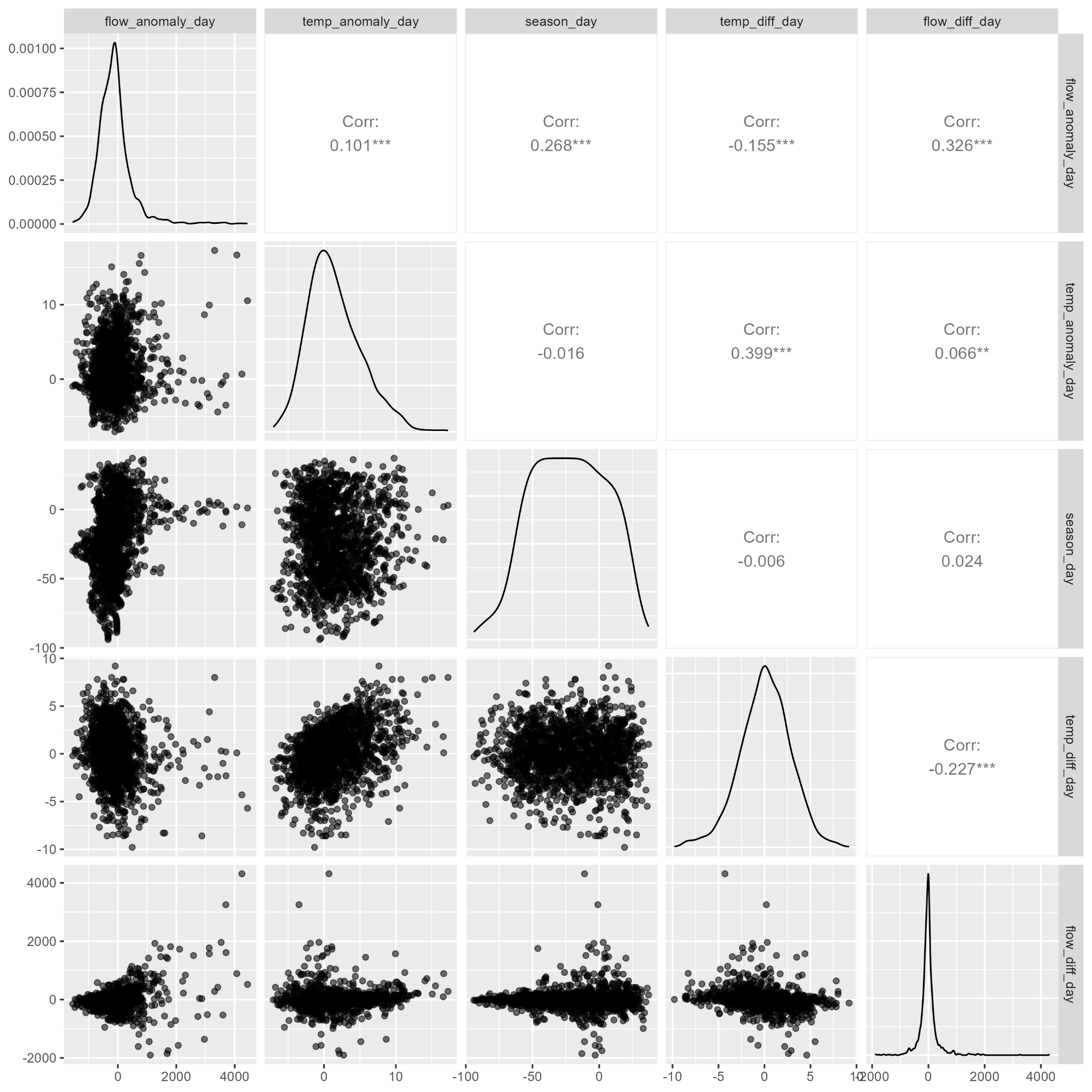


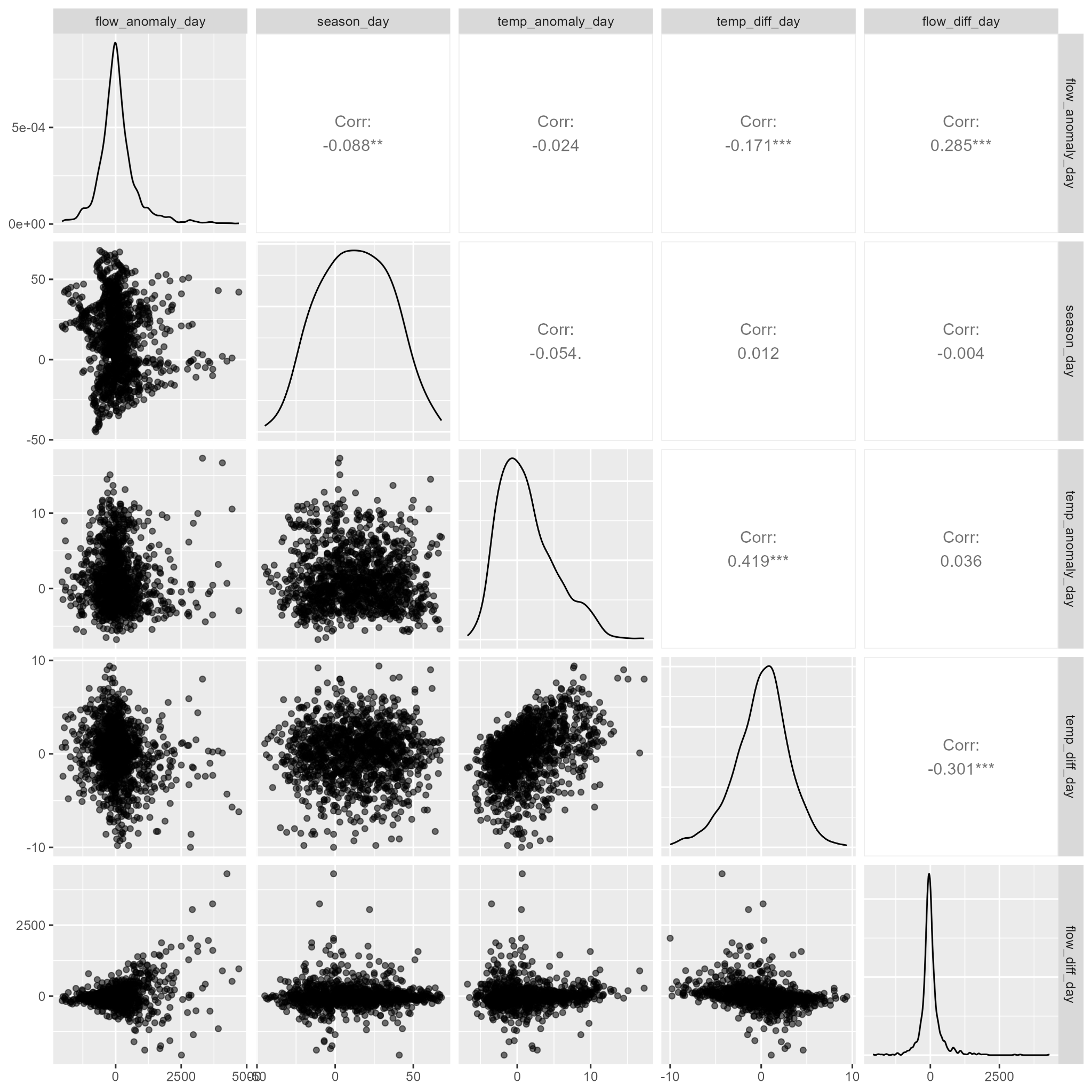


Figure S10 - Correlation between all the environmental variables in the Puyallup River for the day of year 130-218 included in the Chinook model (top) and for day of year 90-160 included in the coho model(bottom). The asterisks denote significance. The diagonal shows the probability density functions for each variable.

| **Chinook** | | **Coho** | |
| --- | --- | --- | --- |
| **Error structure** | $\Delta AICc$ | **Error structure** | $\Delta AICc$ |
| Equal | 0 | Equal | 2 |
| Unequal | 1 | Unequal | 0 |

Table S7 - Model selection with different error structure for day and night for Chinook salmon and coho salmon in the Puyallup River.

|  | |  | |
| --- | --- | --- | --- |
|  |  |  |  |
|  |  |  |  |
|  |  |  |  |
|  |  |  |  |
|  |  |  |  |
|  |  |  |  |

| Season | Temperature difference | Flow anomaly | Flow difference | Temperature anomaly | Hatchery difference day | Hatchery difference night | $\Delta AICc$ |
| --- | --- | --- | --- | --- | --- | --- | --- |
| 0.01 | NA | -0.03 | 0.24 | 0.04 | 0.2 | 0.28 | 0 |
| 0.01 | -0.02 | -0.03 | 0.24 | 0.04 | 0.2 | 0.28 | 0.86 |
| NA | NA | -0.03 | 0.24 | 0.04 | 0.2 | 0.28 | 5.13 |
| NA | -0.02 | -0.03 | 0.24 | 0.04 | 0.2 | 0.29 | 5.47 |
| NA | NA | NA | 0.22 | 0.03 | 0.2 | 0.28 | 24.93 |
| 0.01 | NA | NA | 0.21 | 0.03 | 0.2 | 0.28 | 25.5 |
| NA | -0.01 | NA | 0.22 | 0.03 | 0.2 | 0.28 | 26.3 |
| 0.01 | -0.01 | NA | 0.21 | 0.03 | 0.2 | 0.28 | 27.08 |
| 0.01 | NA | -0.02 | 0.25 | NA | 0.21 | 0.29 | 30.64 |
| NA | NA | -0.02 | 0.25 | NA | 0.21 | 0.29 | 31.83 |

Table S9 - Estimates of the covariates from the top ten models in the model selection with all combinations of uncorrelated covariates for Chinook salmon in the Puyallup River.

| Season | Temperature difference | Flow anomaly | Temperature anomaly | Flow difference | Hatchery difference day | Hatchery difference night | $\Delta AICc$ |
| --- | --- | --- | --- | --- | --- | --- | --- |
| 0.03 | NA | -0.02 | 0.02 | 0.23 | 0.21 | 0.13 | 0 |
| 0.03 | -0.01 | -0.02 | 0.02 | 0.23 | 0.21 | 0.13 | 1.92 |
| 0.03 | NA | -0.02 | NA | 0.24 | 0.21 | 0.13 | 3.86 |
| 0.03 | 0.01 | -0.02 | NA | 0.24 | 0.21 | 0.13 | 5.77 |
| 0.03 | NA | NA | 0.02 | 0.21 | 0.21 | 0.13 | 12.1 |
| 0.03 | 0 | NA | 0.02 | 0.21 | 0.21 | 0.13 | 14.1 |
| 0.03 | NA | NA | NA | 0.22 | 0.21 | 0.14 | 15.5 |
| 0.03 | 0.01 | NA | NA | 0.22 | 0.21 | 0.14 | 17.34 |
| NA | NA | -0.03 | 0.01 | 0.24 | 0.21 | 0.14 | 37 |
| NA | NA | -0.03 | NA | 0.25 | 0.21 | 0.14 | 38.61 |

Table S10 - Estimates of the covariates from the top ten models in the model selection with all combinations of uncorrelated covariates for coho salmon in the Puyallup River.

| **Chinook** | | **Coho** | |
| --- | --- | --- | --- |
| **Variable** | **Relative Importance** | **Variable** | **Relative Importance** |
| Flow difference | 1 | Flow difference | 1 |
| Hatchery difference | 1 | Hatchery difference | 1 |
| Temperature anomaly | 1 | Season | 1 |
| Flow anomaly | 0.99 | Flow anomaly | 0.99 |
| Season | 0.92 | Temperature anomaly | 0.87 |
| Temperature difference | 0.39 | Temperature difference | 0.27 |

Table S11 - Relative variable importance for all variables used in the model selection process for Chinook and coho salmon in the Puyallup River.


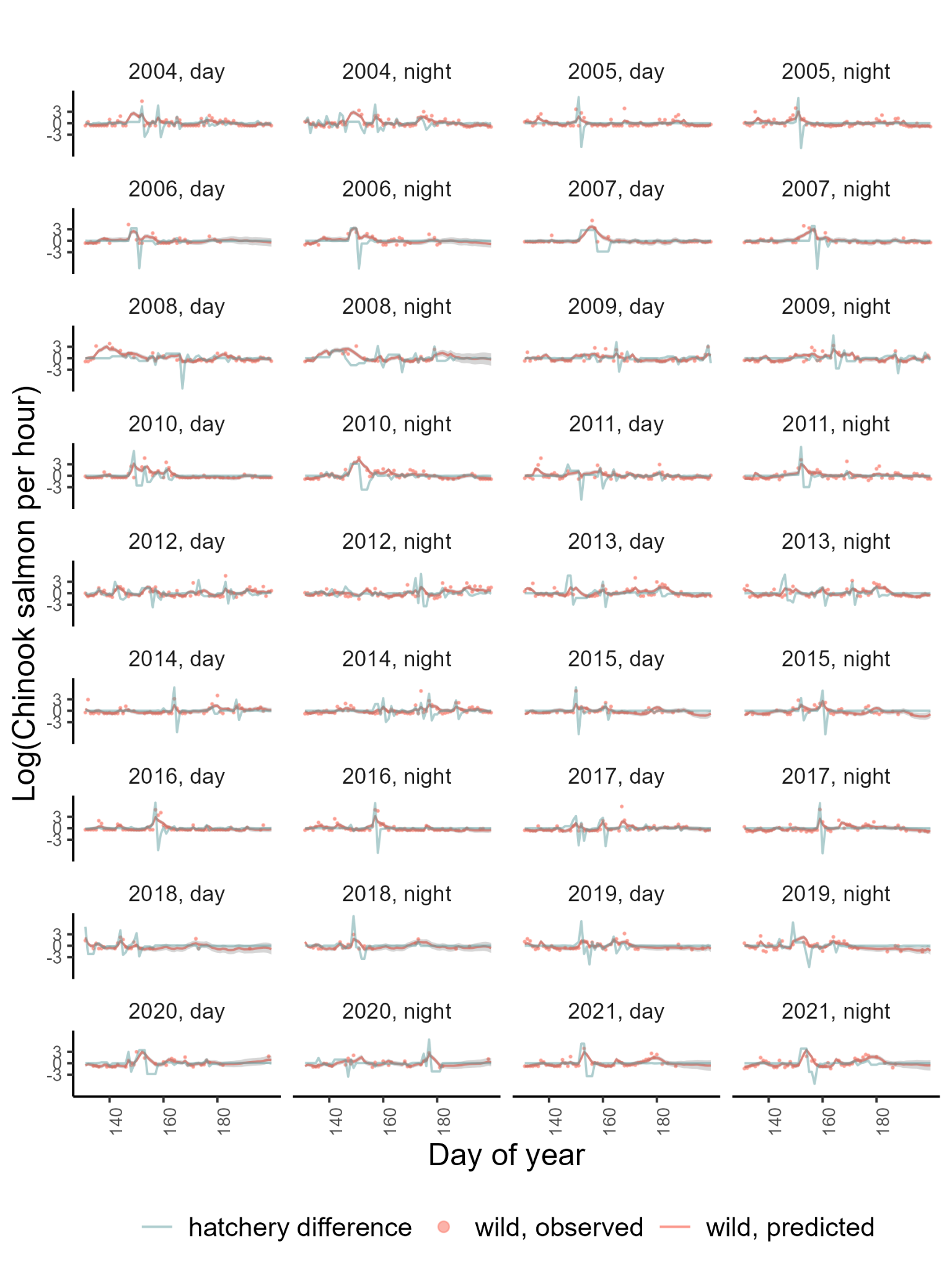


Figure S11 - Model estimates and observations of wild Chinook salmon and observations of hatchery Chinook salmon in the Puyallup River.


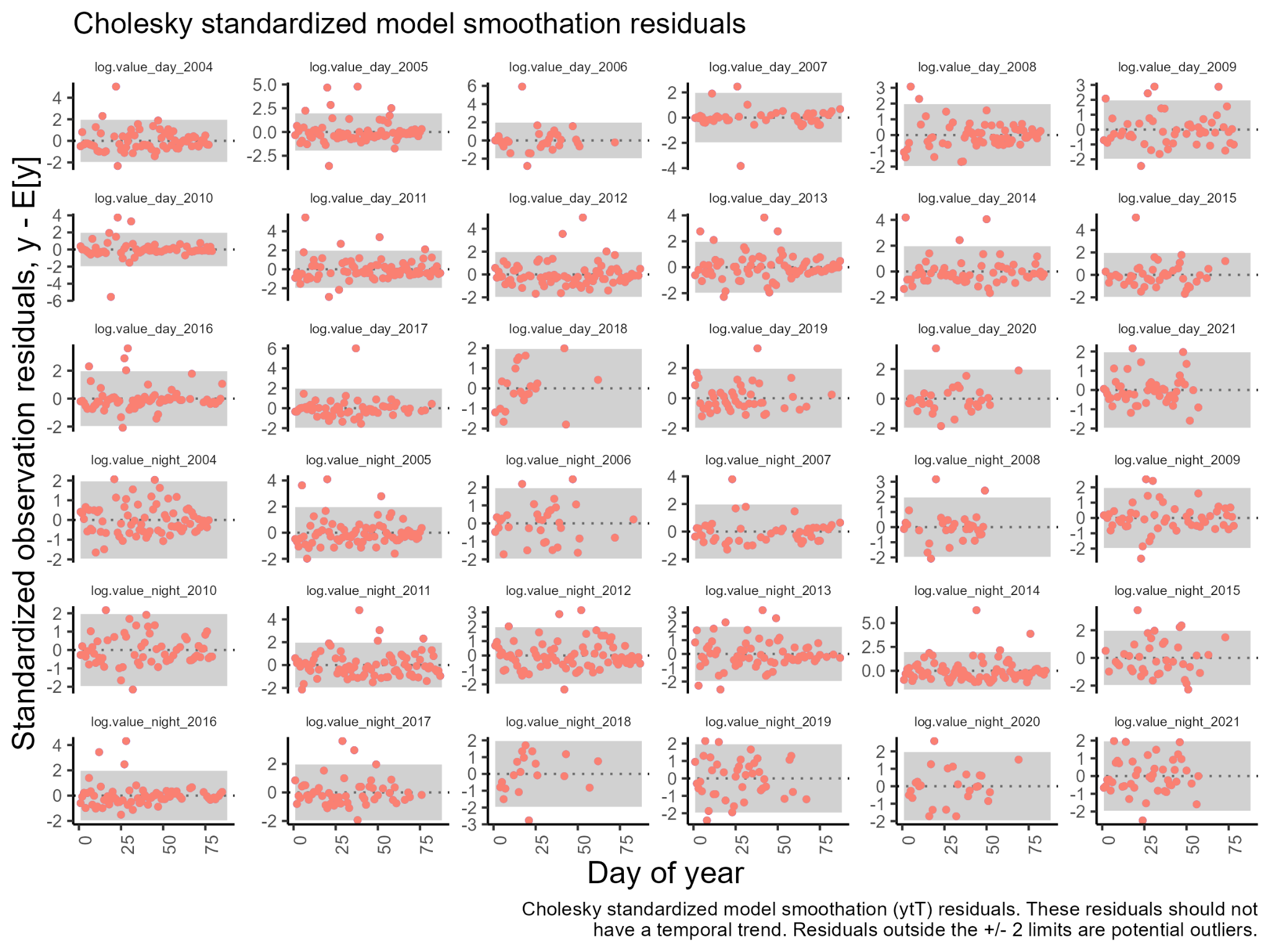


Figure S12 - Model residuals from the best model for Chinook salmon in the Puyallup River.


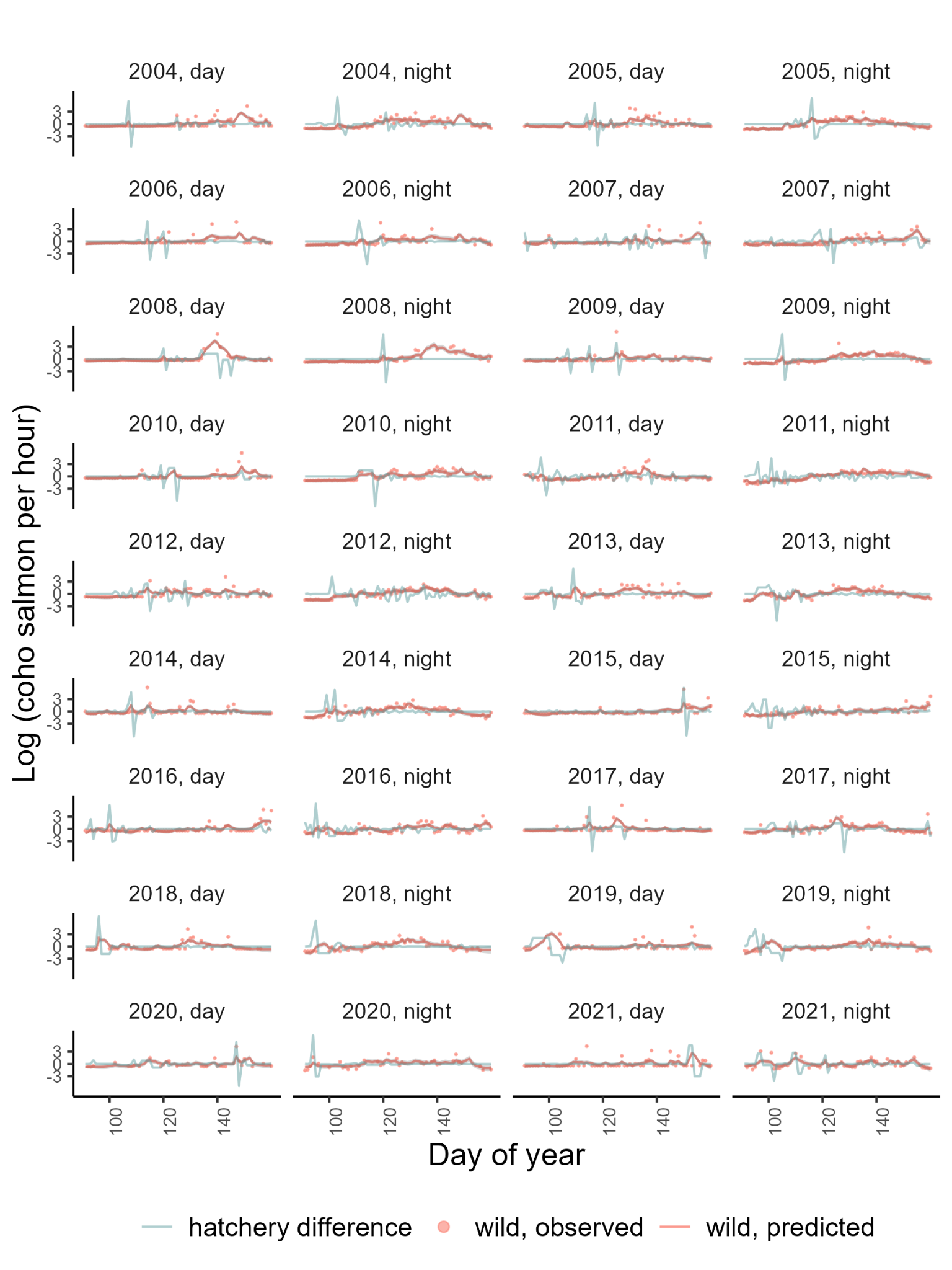


Figure S13 - Model estimates and observations of wild coho salmon and observations of hatchery coho salmon in the Puyallup River.


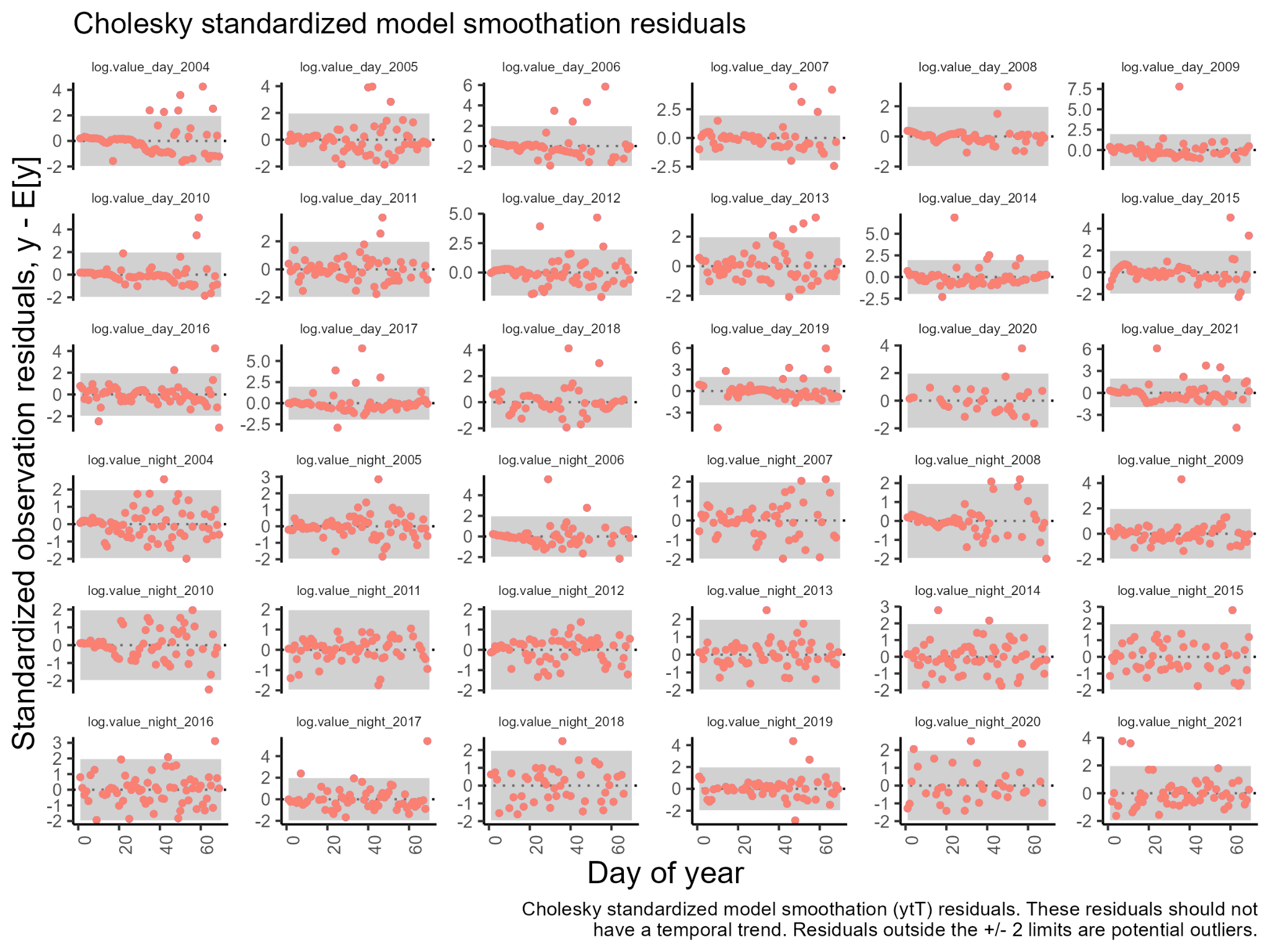


Figure S14 - Model residuals from the best model for coho salmon in the Puyallup River.

### Skagit River

The Skagit River is a 240-km river that originates in the Canadian Cascades and drains into the Puget Sound. Several dams regulate the flow of the river. The Skagit River supports all five species of Pacific Salmon as well as Steelhead and Cutthroat. WDFW operates a screw trap and a scoop trap on the mainstem of the Skagit River (48.4451, -122.3251), 27 km upstream of the river mouth. The traps catch hatchery salmon released from Countyline Ponds, Baker Lake Hatchery, and Marblemount Hatchery.


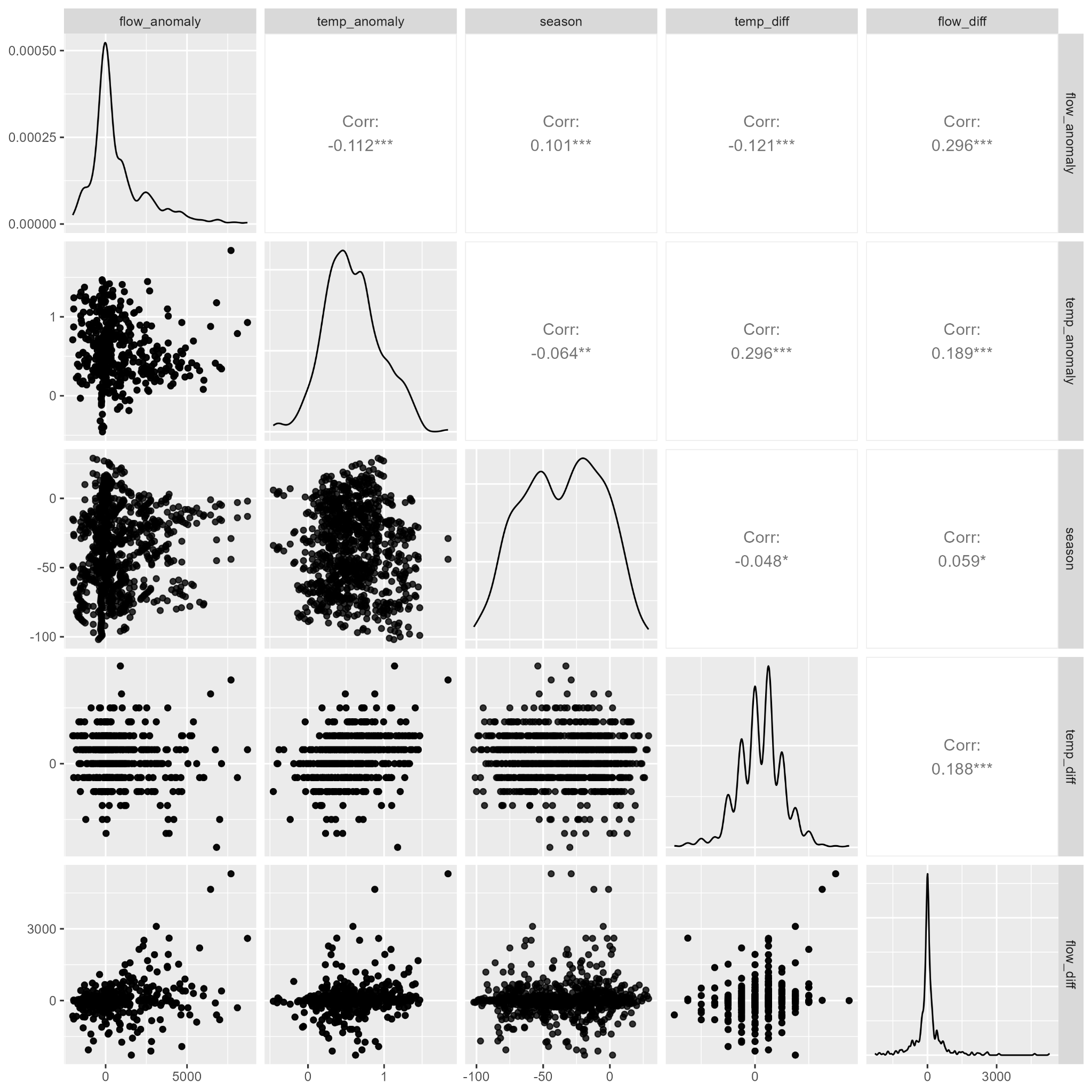


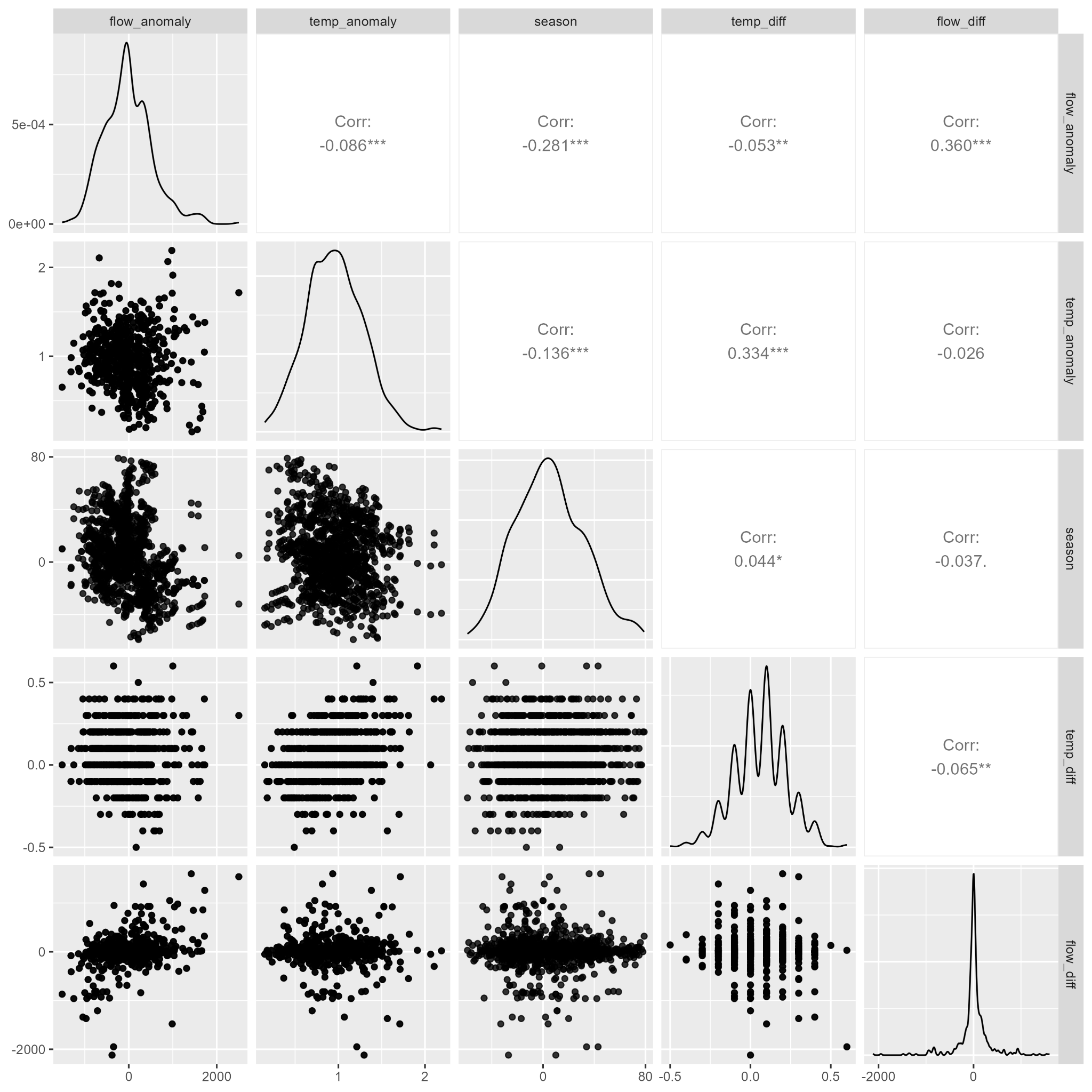


Figure S15 - Correlation between all the environmental covariates in the Skagit River for the day of year 150-189 included in the Chinook model (top) and for day of year 100-150 included in the coho model(bottom). The asterisks denote significance. The diagonal shows the probability density functions for each variable.

|  | |  | |
| --- | --- | --- | --- |
|  |  |  |  |
|  |  |  |  |
|  |  |  |  |

| **Chinook** | | **Coho** | |
| --- | --- | --- | --- |
| **Variable** | **Relative Importance** | **Variable** | **Relative Importance** |
| Season | 1 | Season | 1 |
| Hatchery | 1 | Hatchery | 1 |
| Flow difference | 0.46 | Flow anomaly | 0.94 |
| Temperature anomaly | 0.33 | Temperature difference | 0.39 |
| Flow anomaly | 0.28 | Temperature anomaly | 0.28 |
| Temperature difference | 0.27 | Flow difference | 0.26 |

Table S13 - Relative variable importance for all variables used in the model selection process for Chinook salmon and coho salmon in the Skagit.

| Season | Temperature difference | Flow anomaly | Flow difference | Temperature anomaly | Hatchery difference | $\Delta AICc$ |
| --- | --- | --- | --- | --- | --- | --- |
| 0.05 | NA | NA | NA | NA | 0.29 | 0 |
| 0.05 | NA | NA | 0.03 | NA | 0.29 | 0.37 |
| 0.05 | NA | NA | 0.03 | -0.01 | 0.28 | 1.55 |
| 0.05 | NA | 0.01 | NA | NA | 0.29 | 1.71 |
| 0.05 | NA | NA | NA | -0.01 | 0.28 | 1.74 |
| 0.05 | 0.01 | NA | NA | NA | 0.29 | 2.11 |
| 0.05 | NA | 0 | 0.02 | NA | 0.29 | 2.43 |
| 0.05 | 0 | NA | 0.03 | NA | 0.29 | 2.5 |
| 0.05 | 0.01 | NA | 0.03 | -0.02 | 0.29 | 3.45 |
| 0.05 | NA | 0.01 | NA | -0.01 | 0.28 | 3.54 |

Table S14 - Estimates of the covariates from the top ten models in the model selection with all combinations of uncorrelated covariates for Chinook salmon in the Skagit River.

| Temperature difference | Flow anomaly | Flow difference | Temperature anomaly | Season | Hatchery difference | $\Delta AICc$ | $\Delta AICc$ |
| --- | --- | --- | --- | --- | --- | --- | --- |
| NA | -0.03 | NA | NA | 0.04 | 0.15 | 0 | 0 |
| 0.02 | -0.03 | NA | NA | 0.04 | 0.15 | 0.86 | 0.02 |
| NA | -0.03 | NA | 0.01 | 0.04 | 0.15 | 1.86 | 0.2 |
| NA | -0.03 | 0 | NA | 0.04 | 0.15 | 2.12 | 0.71 |
| 0.02 | -0.03 | NA | 0 | 0.04 | 0.15 | 2.96 | 1.42 |
| 0.02 | -0.03 | 0 | NA | 0.04 | 0.15 | 2.98 | 1.49 |
| NA | -0.03 | 0 | 0.01 | 0.04 | 0.15 | 3.98 | 1.57 |
| 0.02 | -0.03 | 0 | 0 | 0.04 | 0.15 | 5.08 | 1.57 |
| NA | NA | NA | NA | 0.05 | 0.15 | 6 | 1.63 |
| 0.02 | NA | NA | NA | 0.05 | 0.15 | 6.7 | 1.63 |

Table S15 - Model selection with all combinations of uncorrelated covariates for coho salmon in the Skagit River.


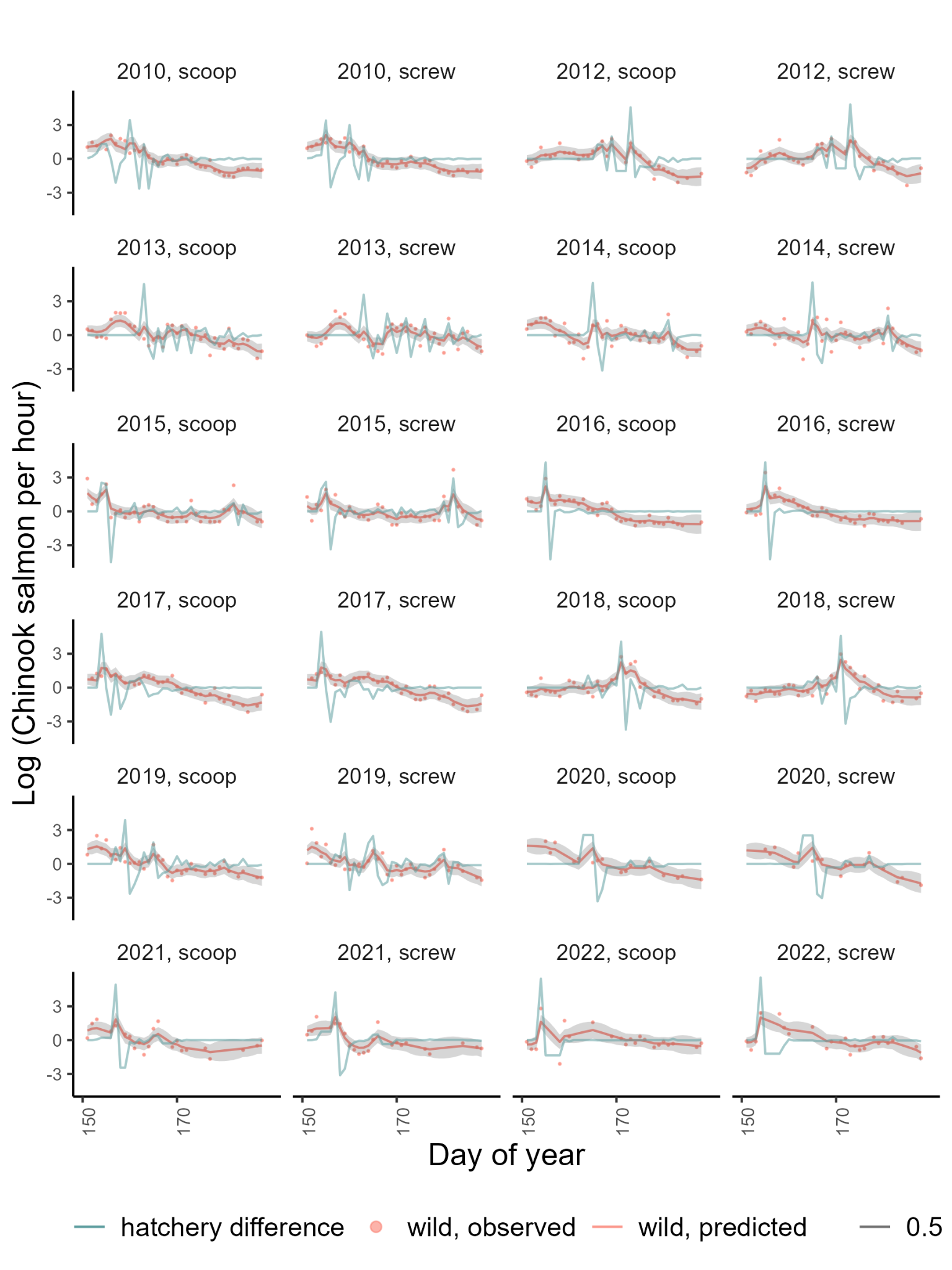
Figure S16 - Model estimates and observations of wild Chinook salmon and observations of hatchery Chinook salmon in the Skagit River.


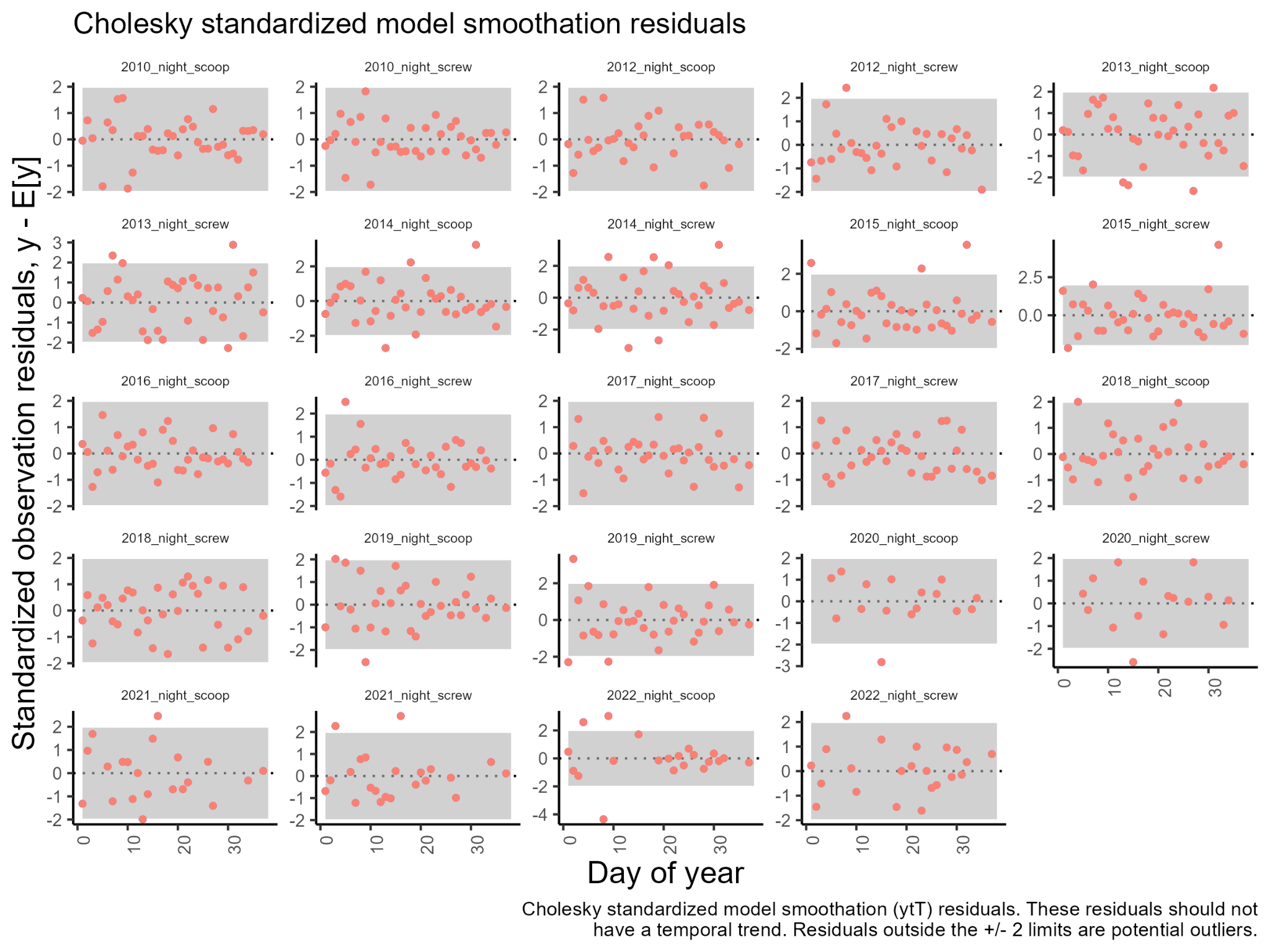


Figure S17 - Model residuals from the best model for Chinook salmon in the Skagit River.


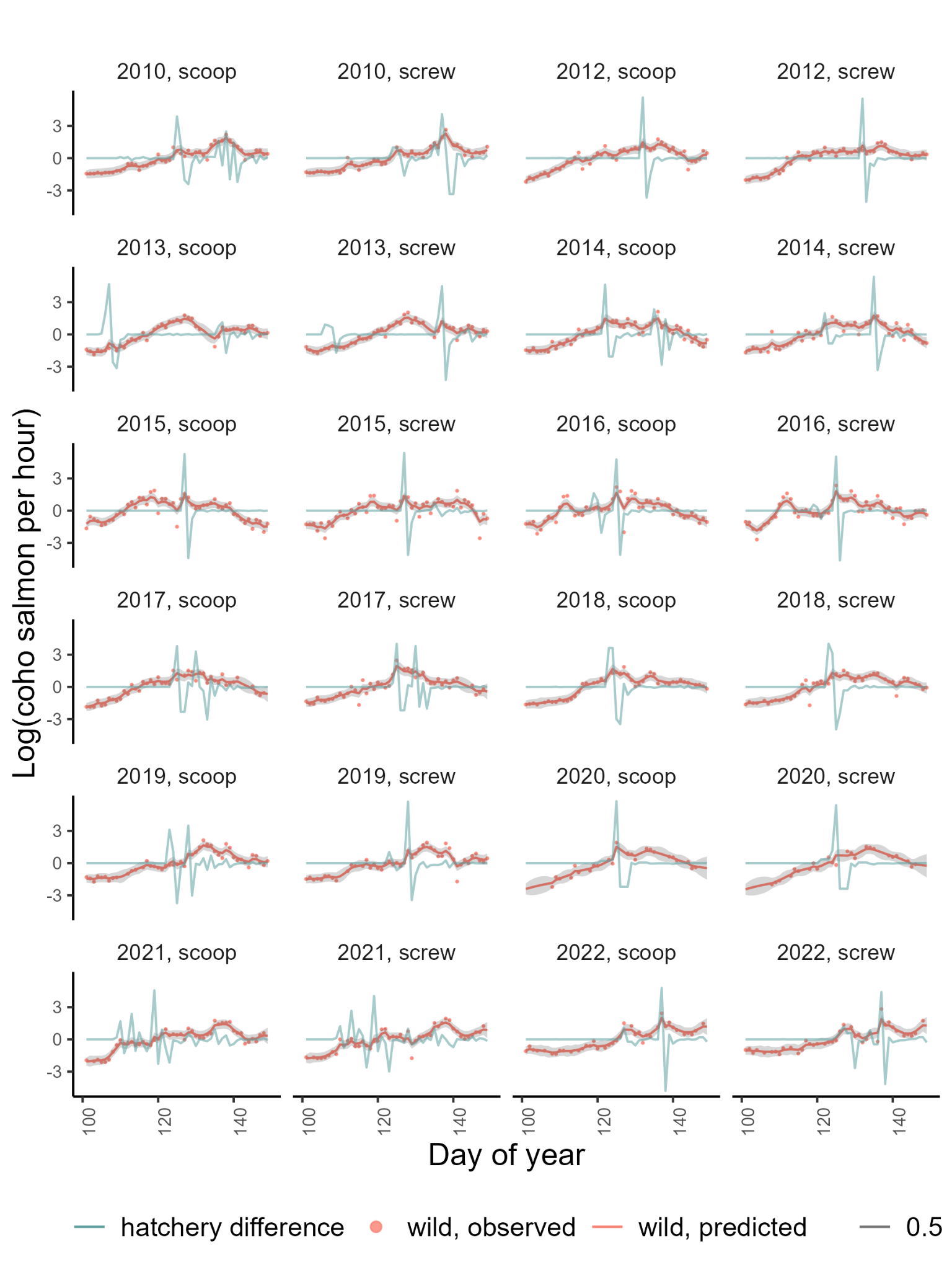


Figure S18 - Model estimates and observations of wild coho salmon and observations of hatchery coho salmon in the Skagit River.


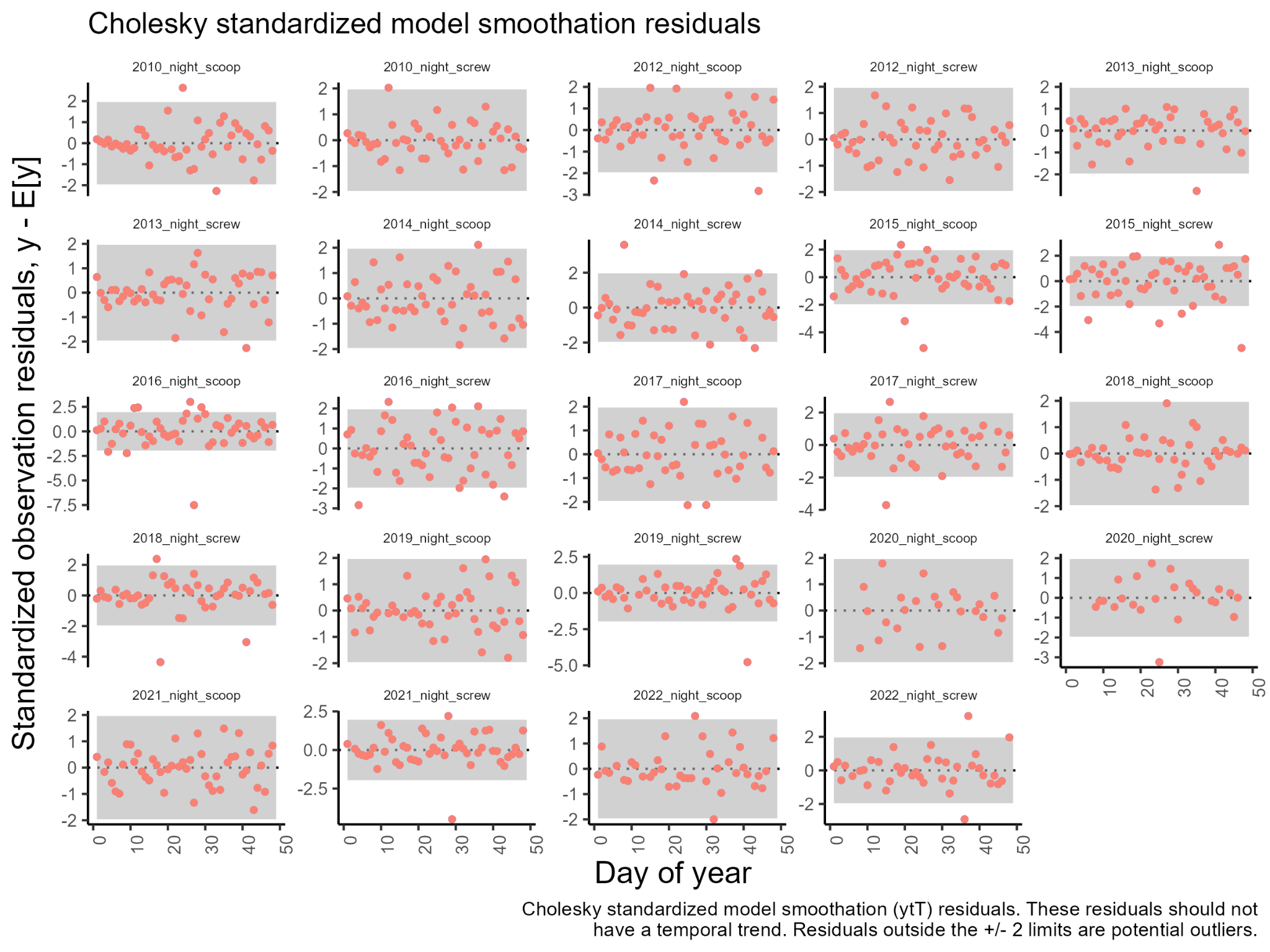


Figure S19 - Model residuals from the best model for coho salmon in the Skagit River.

| Species | River | Average number of releases per year | Average total number released per year | Hatcheries |
| --- | --- | --- | --- | --- |
| Chinook | Dungeness | 2 | 147731 | GRAY WOLF R ACCL PD, DUNGENESS HATCHERY, UPR DUNGENESS ACC PD, HURD CR HATCHERY |
| Chinook | Puyallup | 2 | 987909 | VOIGHTS CR HATCHERY, COWSKULL ACCLIM POND |
| Chinook | Skagit | 2 | 848206 | MARBLEMOUNT HATCHERY |
| Coho | Dungeness | 1 | 548141 | DUNGENESS HATCHERY |
| Coho | Puyallup | 2 | 464371 | VOIGHTS CR HATCHERY, COWSKULL ACCLIM POND |
| Coho | Skagit | 3 | 421403 | MARBLEMOUNT HATCHERY, BAKER LK HATCHERY |

Table S16 - Average number of hatchery salmon released, hatchery location names, and average number of hatchery releases for each river-species combination as reported in RMIS

###

### Sensitivity Analysis


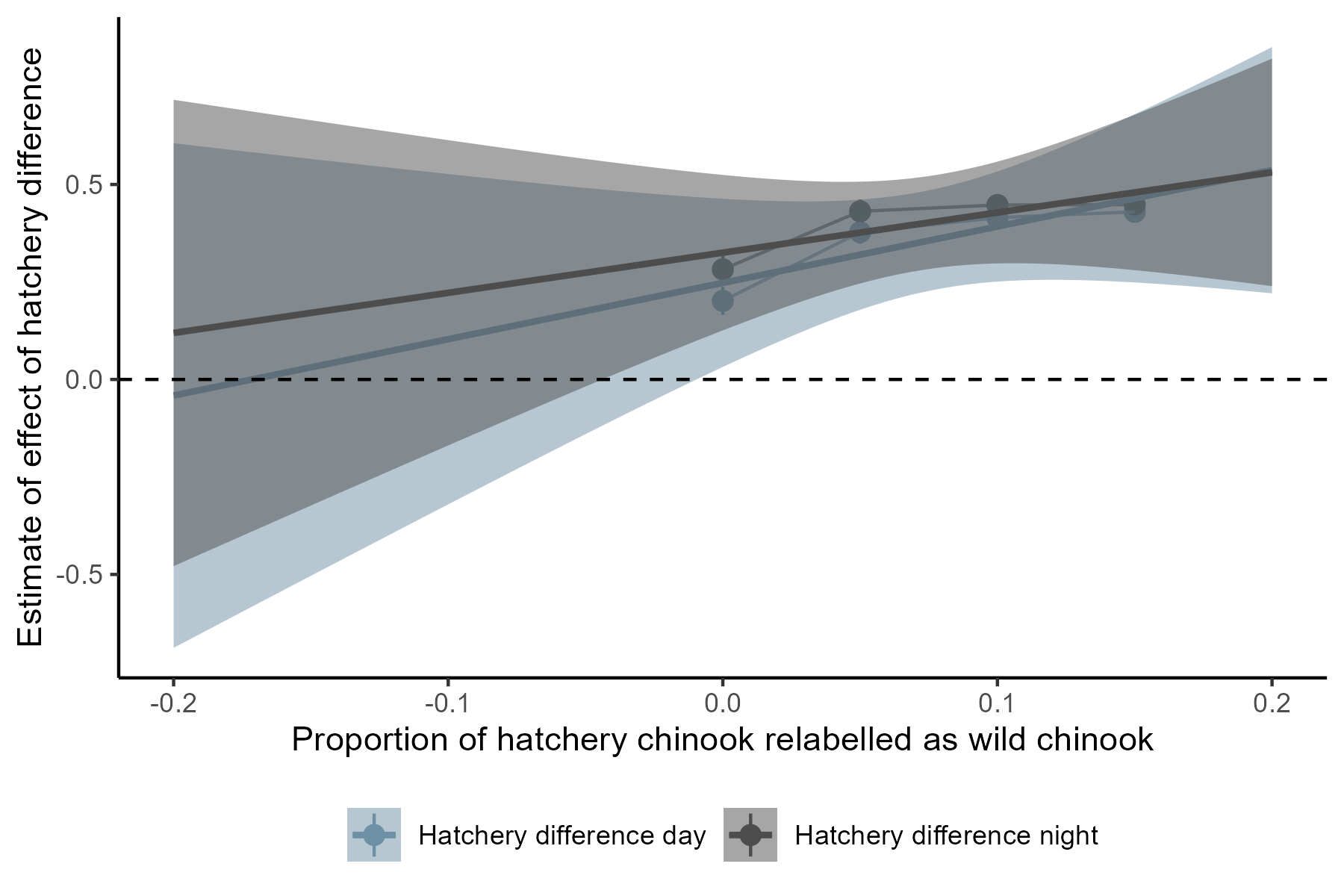


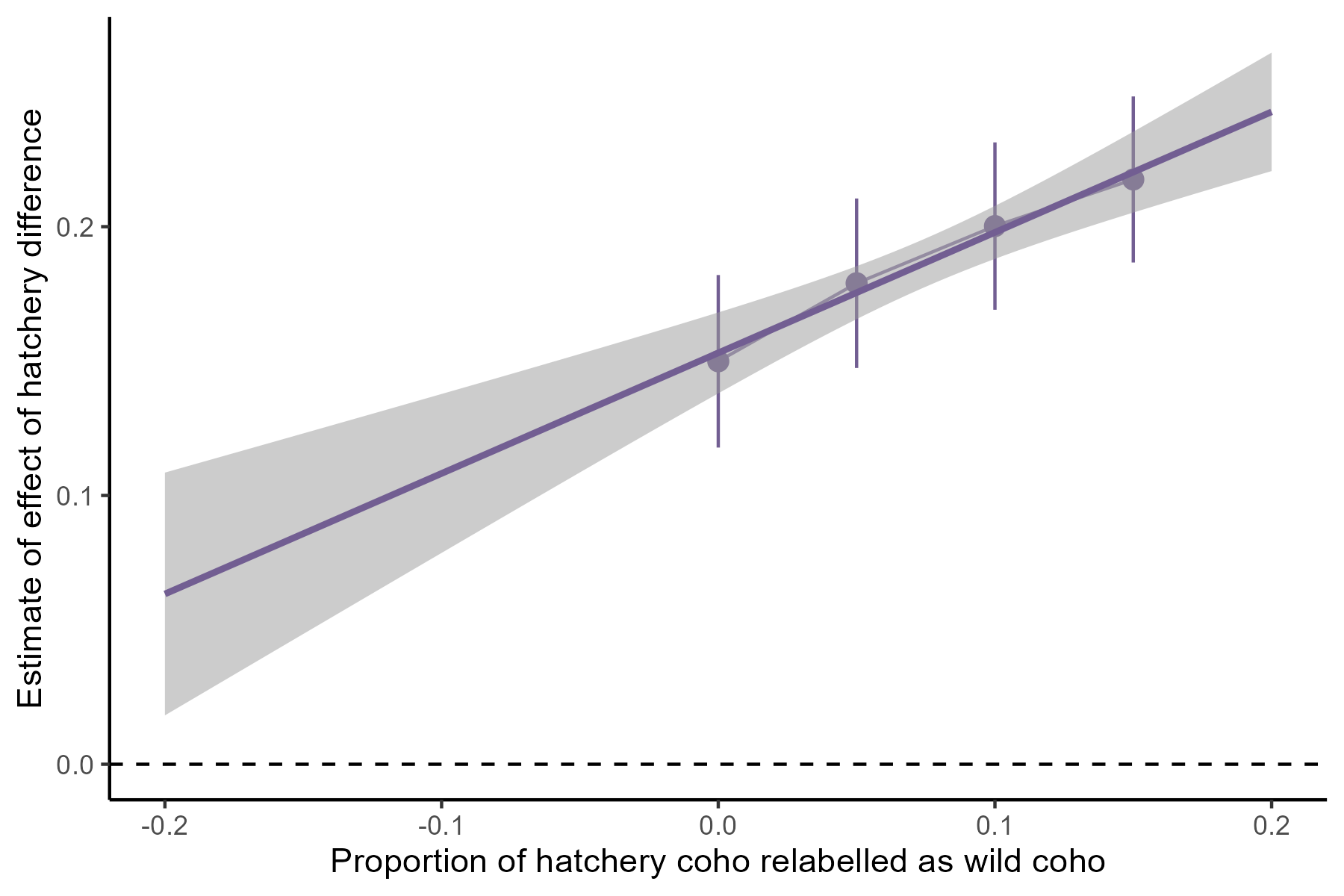


Figure S20 - Sensitivity of the estimate of effect of hatchery difference to the proportion of unmarked hatchery Chinook salmon in the Puyallup river (top) and unmarked hatchery coho salmon in the Skagit River (bottom). To perform this sensitivity analysis we relabelled randomly selected subsets of the known hatchery fish as wild and remeasured the social effect (dots with bars for uncertainty). We did so for various proportions of the hatchery salmon, and fit a linear model to the estimate of effect of hatchery difference as a function of proportion of hatchery salmon relabelled as wild salmon. Using this linear model (line, with shaded 95% uncertainty) we project backward to estimate the effect of unknown unmarked hatchery fish. For Chinook salmon in the Puyallup River where the number of hatchery salmon are disproportionately higher, the results are sensitive to mislabelling hatchery salmon as wild salmon. However, the number of unmarked hatchery salmon were corrected prior to receiving the data. For coho salmon in the Skagit River, even with 20% of the hatchery misassigned as wild fish the social effect is significant. We conclude that the results of coho salmon in the Skagit River are not sensitive to the relabelling.


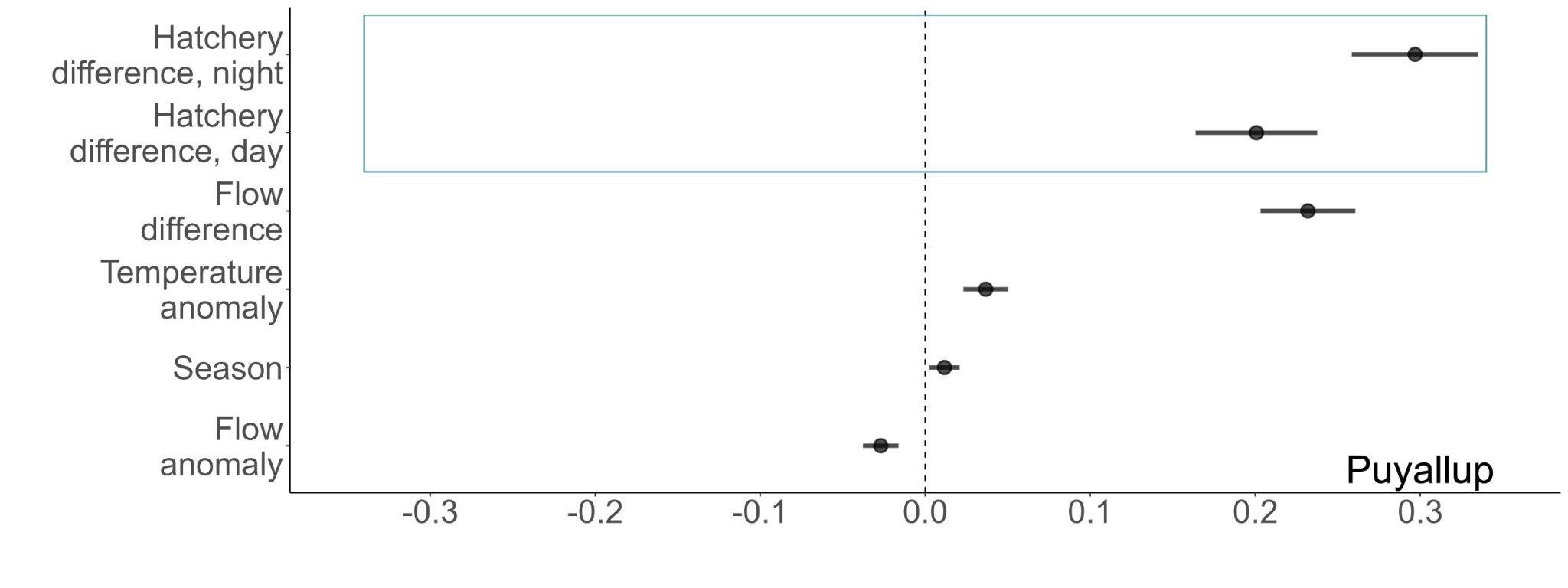


Figure S21 - Estimates of the effect of different covariates in the best MARSS model for the number of wild Chinook salmon in the Puyallup River without 2019 data.


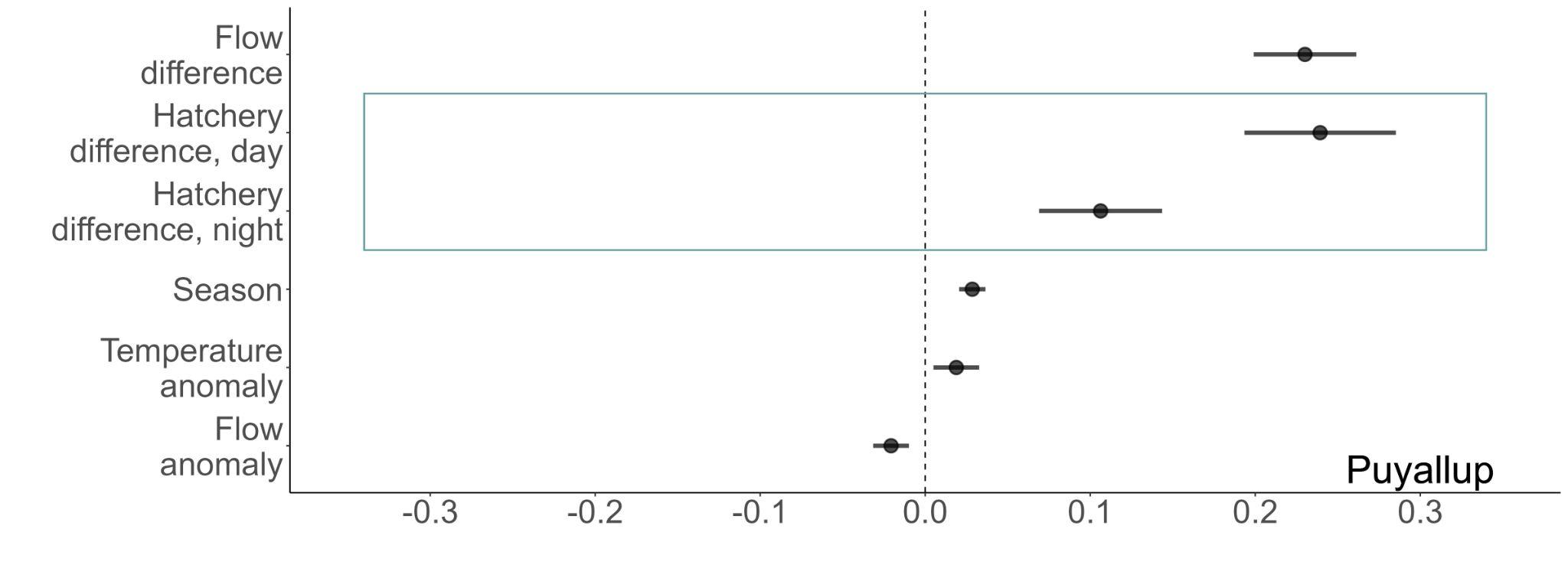


Figure S22 - Estimates of the effect of different covariates in the best MARSS model for the number of wild coho salmon in the Puyallup River without 2021 data.
